# Segmentation and Tracking of Adherens Junctions in 3D for the Analysis of Epithelial Tissue Morphogenesis

**DOI:** 10.1371/journal.pcbi.1004124

**Published:** 2015-04-17

**Authors:** Rodrigo Cilla, Vinodh Mechery, Beatriz Hernandez de Madrid, Steven Del Signore, Ivan Dotu, Victor Hatini

**Affiliations:** 1 Department of Developmental, Molecular & Chemical Biology. Sackler School of Graduate Biomedical Sciences, Tufts University School of Medicine, Boston, Massachusetts, United States of America; 2 Department of Biology, Boston College, Boston, Massachusetts, United States of America; Moffitt Cancer Center, UNITED STATES

## Abstract

Epithelial morphogenesis generates the shape of tissues, organs and embryos and is fundamental for their proper function. It is a dynamic process that occurs at multiple spatial scales from macromolecular dynamics, to cell deformations, mitosis and apoptosis, to coordinated cell rearrangements that lead to global changes of tissue shape. Using time lapse imaging, it is possible to observe these events at a system level. However, to investigate morphogenetic events it is necessary to develop computational tools to extract quantitative information from the time lapse data. Toward this goal, we developed an image-based computational pipeline to preprocess, segment and track epithelial cells in 4D confocal microscopy data. The computational pipeline we developed, for the first time, detects the adherens junctions of epithelial cells in 3D, without the need to first detect cell nuclei. We accentuate and detect cell outlines in a series of steps, symbolically describe the cells and their connectivity, and employ this information to track the cells. We validated the performance of the pipeline for its ability to detect vertices and cell-cell contacts, track cells, and identify mitosis and apoptosis in surface epithelia of *Drosophila* imaginal discs. We demonstrate the utility of the pipeline to extract key quantitative features of cell behavior with which to elucidate the dynamics and biomechanical control of epithelial tissue morphogenesis. We have made our methods and data available as an open-source multiplatform software tool called TTT (http://github.com/morganrcu/TTT)

## Author Summary

Epithelia are the most common tissue type in multicellular organisms. Understanding processes that make them acquire their final shape has implications to pathologies such as cancer progression and birth defects such as spina bifida. During development, epithelial tissues are remodeled by mechanical forces applied at the Adherens Junctions (AJs). The AJs form a belt-like structure below the apical surface that functions to both mechanically link epithelial cells and enable cells to remodel their shape and contacts with their neighbors. In order to study epithelial morphogenesis in a quantitative and systematic way, it is necessary to measure the changes in the shape of the AJs over time. To this end we have built a complete computational pipeline to process image volumes generated by laser scanning confocal microscopy of epithelial tissues where the AJs have been marked with AJ proteins tagged with GFP. The system transforms input voxel intensity values into a symbolic description of the cells in the tissue, their connectivity and their temporal evolution, including the discovery of mitosis and apoptosis. As a proof of concept, we employed the data generated by our system to study aspects of morphogenesis of the *Drosophila* notum.

## Introduction

Epithelial cells form cohesive sheets of cells that play diverse structural and functional roles in multicellular organisms such as the covering of internal and external surfaces, compartmentalization of the body into discrete organs, and the regulation of surface and trans-epithelial transport. The formation of structurally and functionally distinct embryonic structures requires that epithelial tissues change shape during development in a process called epithelial morphogenesis. A range of cellular behaviors drives these epithelial tissue shape changes, including cell shape change, rearrangements of cell-cell contacts, migration, proliferation, and programmed cell death. These behaviors, in turn, depend on intracellular molecular dynamics that allow cells to generate and transmit mechanical forces to one another, while maintaining epithelial cohesion [1, 2]. This dual requirement is fulfilled by the adherens junction (AJ), a specialized protein complex that links epithelial cells together. The AJs form a planar belt-like structure below the apical surface of the epithelium composed primarily of the single pass adhesion protein E-cadherin (E-cad) and associated proteins. The extracellular domain of E-cad forms trans homo-dimers to promote cell adhesion. The intracellular domain of E-cad associates with the force-generating actomyosin cytoskeleton and functions as a site for the transmission of mechanical forces that can remodel cell-cell contacts and cell shape by influencing the dynamics of the AJs themselves [3].

Despite advances in understanding the roles of the AJs and their regulators in controlling epithelial morphogenesis, we still do not understand how intracellular forces and cell behaviors coordinate at the tissue level to drive epithelial morphogenesis. Specifically, cell shape may be controlled by either autonomous or non-autonomous behaviors or forces, while the interaction between such local dynamics can lead to emergent effects on cell or tissue morphology. Live imaging of cell and molecular dynamics using fluorescently-tagged proteins is a key method to investigate these processes [4]. However, to fully leverage these experimental methods, quantitative approaches to automatically identify, track and interrelate molecular, cell and tissue level dynamics are required. The analysis of this quantitative information could then suggest molecular, cellular, and tissue level mechanisms that drive morphogenetic processes, and guide experimental approaches to test these possible mechanisms [5–9].

Several methods for segmentation and tracking of epithelial cells have been developed. These methods are based on the detection of the AJs in projections of 3D information into 2D planes. These methods provide approximations of epithelial shape but often lead to inaccurate representations of cell shape, especially in curved regions of epithelial sheets. The projection of an image volume into a 2D plane also increases the image noise, which may interfere with image preprocessing and quality of segmentation. Therefore, to circumvent these problems and provide a more accurate representation of epithelial cells, methods to segment and track the AJs in 3D need to be developed.

Most methods for the segmentation and tracking of biological entities in 4D microscopy data are designed to establish the identity of particle-like objects such as nuclei [10–12], organelles or molecular assemblies [13, 14]. These methods consider objects as particles moving independently without restrictions imposed by cell packing in an epithelial sheet, and thereby do not model cell shape and cell-cell contact dynamics. Only a few methods have been developed for the segmentation and tracking of cells in tissues. A common approach is to perform a dual analysis of multichannel time lapse images labeled for both nuclei and membrane. Locations of cell nuclei are first identified and later employed to find the location of the cell membrane. Using this approach Bourgine et al. [15, 16] have built a Partial Differential Equation framework to filter image noise, segment cell nuclei, locate the cell membrane and model the evolution of cell shapes to track the cells. A similar scheme has been developed by Luengo-Oroz et al. who employed a 4D morphological structuring element framework to denoise image volumes and locate cell nuclei, and a viscous watershed algorithm to infer cell nuclei locations as seeds for detecting cell extent properties [17]. This process is performed in 4D, implicitly tracking the cells. The same group has developed an alternative 3D based scheme to first identify nuclei, employ the viscous watershed method to determine cell extents and then track the cells and identify mitotic events [18].

Recently, several methods have been developed to detect the plasma membrane in tissues labeled with a membrane marker only. Mosaliganti et al. have built the ACME system to detect the planar structure of the cell membrane, and employed it to segment the spatial extent of cells in zebrafish embryos [19]. The EDGE system has been developed to segment and track epithelial cells in *Drosophila* embryos using a spatial alignment of 2D slices of a membrane marker to reconstruct the structure of the plasma membrane. The 2D slices are first denoised and thresholded, and then polygonal approximations are fitted to the segmented regions and stacked in 3D. Finally, the cells are tracked based on a custom polygon matching method [8]. A new version of the software called EDGE4D was recently published [20], employing fluorescently labeled nuclei in the cell segmentation.

All the previous methods focused on recovering the full spatial extent of the cells delimited by membranes. However, our final goal is to study the role of molecular forces remodeling AJs so we sought to develop methods to segment and track cells in epithelial tissues using only AJ markers. Although the AJs arrange in a belt-like structure, methods to detect this structure in 3D have not been reported. To the best of our knowledge, segmentation and tracking of AJs have only been done using 2D max-intensity projections of confocal time lapse data. Watershed algorithms are employed by Packing Analyzer [6] and by SeedWater Segmenter [21] to segment the AJs in 2D. A 2D simplification of the 3D Partial Differential Equation framework introduced in [15] has been reported in [22]. AJs assume the role of membranes when computing cell extent properties from cell nuclei. All the above methods are not easily generalized to 3D because the AJs do not surround the cells in 3D as they do in 2D.

Successful segmentation of AJs and tracking of cells in confocal image volumes require solutions to several challenges that arise from the dynamic properties of the cells and the fluorescent E-cad∷GFP reporter used to highlight the AJs. In morphogenetic epithelia, E-cad molecules are constitutively endocytosed and recycled back to the cell surface providing epithelial cells with the plasticity to dynamically rearrange cell shape and cell-cell contacts [23–25]. As a result, E-cad is highly enriched in endocytic vesicles that can produce false positive membrane detections. At the cell surface, the distribution of E-cad is not always uniform increasing the complexity of detecting the AJs as a continuous structure. Certain cell types such as sensory cells accumulate high levels of E-cad thus increasing the complexity of segmenting these cells. Membrane detection methods have to distinguish between membrane and cytoplasmic signal, bridge discontinuities in signal distribution, and tolerate high signal intensity in certain locations.

During epithelial morphogenesis, the cells grow or shrink, narrow or elongate, move and exchange neighbors, divide and die. Cell tracking methods in epithelial tissues need to identify cells as they change shape and contacts with their neighbors. To ensure cell track consistency it is also important to detect dividing cells and properly label the two daughter cells of mitotic events instead of creating a new track for one of them. Similarly, when a cell dies, the event should be properly identified and distinguished from a cell that leaves the scene. Tissue drift causes new cells to enter the scene and others to leave it. The cells that enter and leave the scene should be distinguished from cells that are generated by mitosis and lost by apoptosis, respectively. Tissue drift also introduces a global motion that should be compensated for.

Here we present a novel approach for the automated segmentation and tracking of epithelial cells labeled with genetically encoded E-cad∷GFP using 4D confocal microscopy data of two developing epithelia: the *Drosophila* leg and notum. We chose these tissues for their unique morphological and developmental characteristics. The leg is a tube-like epithelium, which narrows and so elongates dramatically during development, while the epithelium of presumptive joints invaginates by apical constriction [26]. By contrast, the notum is a more planar epithelium that undergoes more subtle morphological changes [27]. The proposed approach to detect the AJs is independent of a prior detection of nuclei. Instead, cells are detected and tracked based on segmentation of the AJs and the determination of the connectivity between cells in the tissue. The methods we developed: 1) directly detect the vertices of the AJs where three or more cells meet in image volumes; 2) compute a planar graph approximation to the AJs network structure; 3) compute cell locations in the tissue and spatial associations between cells; 4) track cells and discover mitotic and apoptotic events. Additionally, we show how the computed motion of cell centroids can be used to describe the global dynamic behavior of a tissue. Finally, we provide a framework to assess the performance of the methods over a range of parameter values.

## Results

### Segmentation and Tracking of Adherens Junctions in 3D

We have developed a system to segment the AJs of epithelial cells highlighted by AJ markers such as E-cad∷GFP and find the correspondence among them in adjacent temporal frames. The system is able to segment and track from just a few to hundreds of cells in 3D under different imaging conditions, including planar epithelial tissues such the *Drosophila* notum or tube-like epithelial tissues such the *Drosophila* leg.

**Fig 1:**
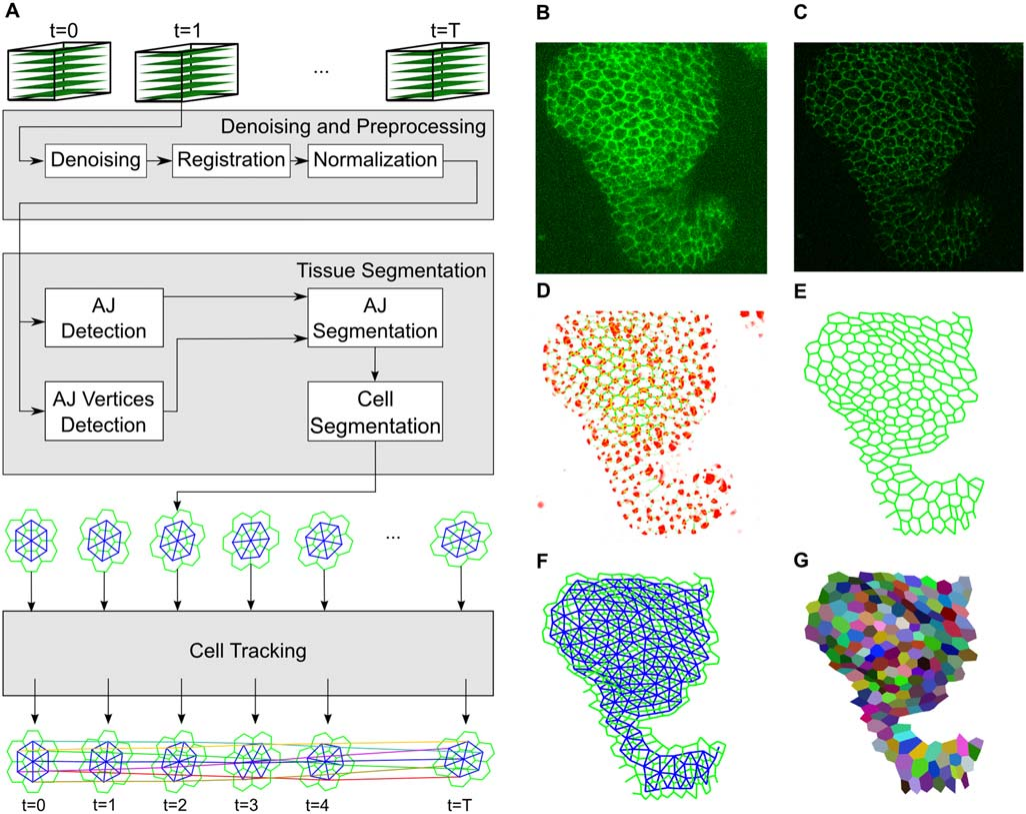
The developed system for the preprocessing, segmentation and tracking of epithelial cells. A) Schematic of the computational pipeline from the acquisition of 3D time lapse data to image preprocessing, cell segmentation and tracking. After segmenting the cells, we define symbolically their structure using a planar graph connecting detected AJ vertices with edges (green). Then we identify the cells in the tissue as the faces of the AJ graph and build the Cell graph to describe cell connectivity (blue). Finally, we establish correspondence between cells among frames (colored lines connecting cell centroids) obtaining cell trajectories. (B-G) Part of an epithelium of a *Drosophila* leg at early pupal stages. This tissue dramatically narrows and elongates at this stage to generate a narrow and hollow cylinder while the epithelium at presumptive joints invaginates. B) Maximum intensity projection of an image stack through the leg epithelium marked with E-cad∷GFP to highlight cell outlines. Distal up, narrow region—presumptive joint; wider regions part of the presumptive segment. C) Projection of the denoised and deconvoluted volume. D) The output of the filters employed to detect AJs (green) and AJ vertices (red). E) AJ graph representing the AJs structure. F) Cell graph representing neighborhood relationships among cells in the tissue. G) Polygonal representation of the cells, colored according to assigned temporal identifiers.

Fig 1A outlines the computational pipeline we developed to preprocess, segment and track epithelial cells. First, confocal time lapses of epithelial tissue morphogenesis (Fig 1B) are acquired. To enhance the representation of the AJs, timelapses are deconvoluted to eliminate both out-of-focus signal, and the Poisson noise introduced by the photodetector during imaging (Fig 1C). If required, the images are locally equalized to obtain an even intensity of the E-cad∷GFP signal employing Contrast Limited Adaptive Histogram Equalization (CLAHE) [28].

Second, the AJs are located in the input volumes and encoded into a spatial graph to mimic their approximate polygon structure. A pair of filters based on the second order spatial derivatives of the image intensity are employed to measure the likelihood of each voxel being part either of a vertex or an edge [29] (Fig 1D). The vertices of the graph are initialized with the local maxima of the output of the vertex detection filter. A level-set based region growing algorithm is employed to establish the connectivity among vertices in the input volume, adding an edge to the AJ graph for each pair of adjacent vertices (Fig 1E). See materials and methods for details.

Third, the cells in the tissue are identified and encoded in the Cell graph. The AJ graph is planar and has a face for each cell. The Cell graph (Fig 1F) is obtained as the dual of the AJ graph, with a vertex for each cell and an edge for each pair of adjacent cells. Each cell is associated with a list of vertices in the AJ graph defining its spatial extent. The spatial moments of this polygonal representation provide a set of spatial features for each cell such as perimeter, area, centroid, size or rotation.

**Fig 2:**
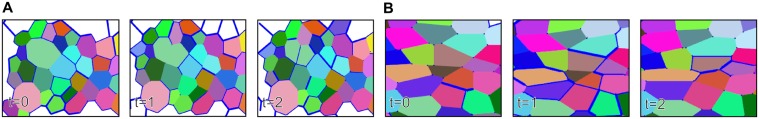
Detection of mitosis and apoptosis events. In addition to cell movements, notum morphogenesis involves extensive cell delamination at the dorsal midline [30] and extensive cell proliferation required for notum expansion [7]. We therefore incorporated into the tracker algorithms to detect mitotic and apoptotic events. A) The large light green cell in the upper left region splits to produce two daughter cells marked by the same color. B) The dark brown cell in the center reduces its area until it disappears.

Finally, the correspondence among cells in adjacent frames is resolved (Fig 1G). To this end we employ a min-cost max-flow cell tracking framework capable of detecting cellular mitosis and apoptosis events [10] (Fig 2). We consider that the detection of these events is as important as the correct association of cells among frames, although their relatively low frequency makes their detection even harder. However, understanding where and when cells proliferate and die is as important as studying how cells change their shape. Our system is able to successfully detect cellular apoptosis as the area of the dying cells tends to vanish and to detect cellular mitosis as the spatial moments of the parent cell are similar to the moment of the union of the daughter cells.

The segmentation and tracking algorithms employed provide reliable outputs with some errors as we show later. We developed tools to fix errors in the output of the vertex location, edge segmentation and cell tracking algorithms (Screenshot shown in S11 Fig). Error correction is done after each step as errors at one step are highly magnified in the next. Error correction ensures that the data to be analyzed accurately reflects the observed data.

### Case Study: Studying Morphogenesis of the *Drosophila* Notum

**Fig 3:**
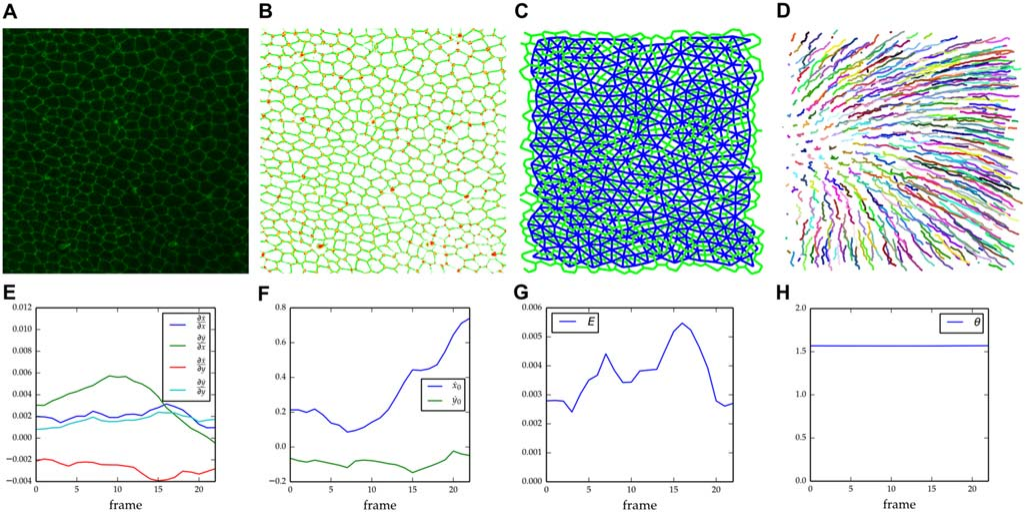
Analysis of *Drosophila* notum morphogenesis. A) A Maximum intensity projection of an image stack through the mid-scutum marked with E-cad∷GFP to highlight cell outlines. Anterior to the right. B) Maximum intensity projection of the output of the filters employed to detect the AJs (green) and AJ vertices (red). C) AJ graph (green) and Cell graph (blue) symbolically represent the cell outlines and their connectivity, respectively. D) Projection of the 3D centroid trajectories recovered after tracking the motion of cells. Note that motion of cell centroid varies depending on the position of the cell across the tissue. E) Evolution of cell strain rate parameters ($\frac{\partial \dot{x}}{\partial x}$, $\frac{\partial \dot{x}}{\partial y}$, $\frac{\partial \dot{y}}{\partial x}$, $\frac{\partial \dot{y}}{\partial y}$) computed from Kalman smoothed trajectories of cell centroids over time. F) Mean velocities (${\dot{x}}_{0}$, ${\dot{y}}_{0}$) of the cell centroid trajectories over time. Time evolution of G) expansion coefficient ℰ, and H) rotation coefficient *θ*. See text for further detail.

We illustrate the usage of the system to study the morphogenesis of a region of a *Drosophila* notum 24 hours after pupariation (apf) exploring the collective cell behaviors that contribute to tissue deformation. The timelapse captured the dynamics of cells in an area around the midline of the *mid-scutum*. We have employed the system to recover AJ graphs and cell graphs (Fig 3B and 3C), identifying the cells in the tissue and establishing the temporal correspondence among them (Fig 3D). The output of the system included some error that we corrected manually with validation tools that we developed to obtain accurate data. A visual inspection of the recovered cell trajectories shown in Fig 3D reveals a velocity gradient, increasing from posterior (left) to anterior (right). To understand the reason for this, we have built a model to quantify the process that contributes to the global deformation of the tissue at this developmental stage.

The strain rate tensor of a vector field describes the instantaneous rate of change of the deformation of a continuous material [31]. Because the epithelium of the notum is relatively planar, we dropped the third dimension of the cell trajectories and we built a strain rate tensor for the tissue, taking the velocity vector field of the cell centroids. The strain rate tensor components ($\frac{\partial \dot{x}}{\partial x}$, $\frac{\partial \dot{x}}{\partial y}$, $\frac{\partial \dot{y}}{\partial x}$, $\frac{\partial \dot{y}}{\partial y}$) and the mean velocities (${\dot{x}}_{0}$, ${\dot{y}}_{0}$) of the vector field are shown in Fig 3E and 3F. Their values confirm that the velocity vectors depend on the position of each cell in the tissue and their signs show they are higher the farther they are from the posterior *scutellum*. We decomposed the strain rate tensor at each frame into their symmetric and antisymmetric components to respectively compute the expansion coefficient ℰ (Fig 3G) and the coefficient of rotation of the tissue *θ* (Fig 3H). The expansion coefficient is always positive as the surface area of the tissue increases during the process. The coefficient of rotation remains almost constant, reporting a global rotation of the tissue of about one degree between frames,very likely resulting from a drift of the tissue in the medium.

Prior to computing the strain rate tensors we have preprocessed the cell centroid trajectories to remove the noise in them employing a Kalman filter [32] (see Materials and Methods). The Kalman filter assumes that cells obey a linear motion model among frames, providing a smoothed estimation of the position and velocity of each cell at each instant.

### Performance Evaluation

We assessed the performance of the algorithms employed to segment the AJs and track the cells in 4 different time lapse data sets. Performance metrics were computed by comparing the outputs of the system to a ground truth, which was established through manual correction of the system output. For the notum dataset we corrected (added, moved or deleted) about 100 vertices for a total of 824 vertices and 60 edges (added or deleted) for a total 1207 it contains. For the leg dataset we corrected 120 vertices for a total of 403 and 93 edges for a total of 586. The aim of this performance evaluation framework is not only to assess the quality of the proposed algorithms but also to provide a comparison framework to assess the quality of future alternatives.

First, we analyzed the performance of the vertex location method. We limited the assessment to the first frame of the notum and leg time lapses. A vertex in the ground truth is considered as detected if the algorithm marks a vertex location closer than $\sqrt{2}$ voxels from the manually annotated position, i.e., a vertex is detected if there is a detection in any of the voxels around it. The vertex location method depends on the interval of spatial scales employed to control the size of the AJ vertices detected and a detection threshold controlling the strength of the detected vertices. The spatial scale interval is easy to set up visually to an acceptable value, so we only assess the performance regarding variations in the vertex detection threshold. See supplementary S4 Fig for guidelines on how to select the proper spatial scales.

**Fig 4:**
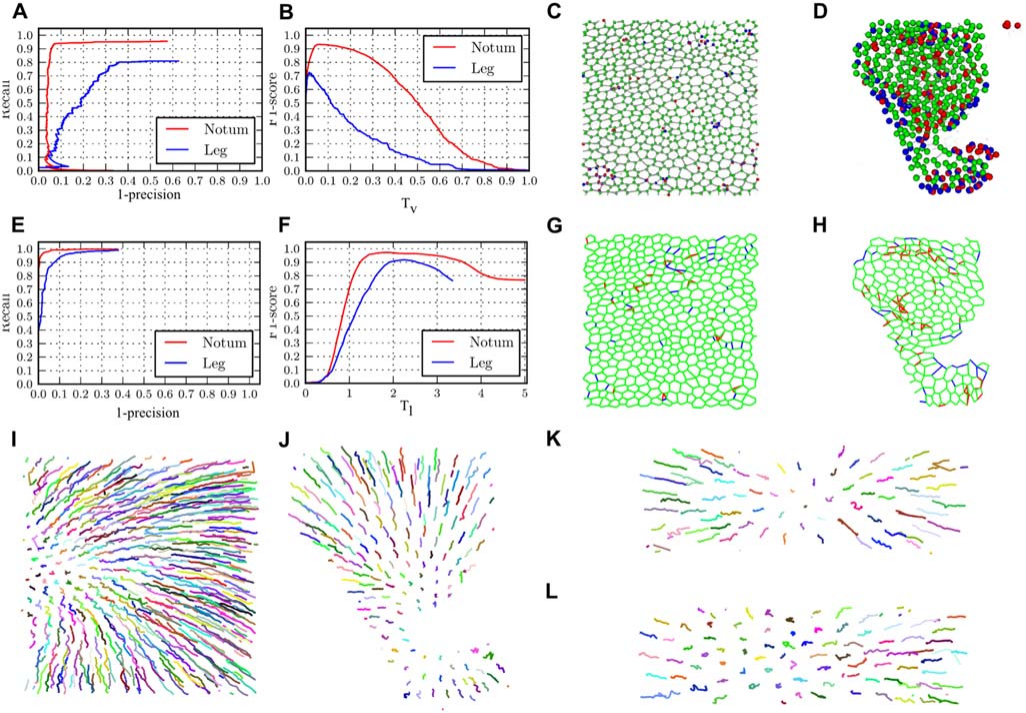
Performance assessment of the automated detection of vertices, cell-cell contacts and cell tracks. A-D) Evaluation of the vertex detector, E-H) edge detector, and I-J) cell tracker. C-D), G-H) Green—true detections, blue—missed detections, red—false detections. A) Precision-Recall curve for AJ vertex detection. B) Variation of the *F*1 score of the vertex detector relative to changes in detection threshold *T*_*V*_. Vertex detection of C) Notum and D) Leg datasets. Vertex location accuracy highly depends on properly tuning up *T*_*v*_. E) Precision-Recall curve for AJ edge detection. F) Variation of the *F*1 score relative to changes in propagation threshold *T*_*E*_. Edge detection in G) Notum and H) Leg datasets. Edge detection is more robust than vertex detection. I), J), K) and L) 2D projection of the trajectories respectively found for the cells in E) Notum, F) Leg, G) Mitosis in notum and H) Apoptosis in notum datasets. The system recovers accurate cell trajectories in different scenarios.

Fig 4A shows Precision-Recall curves generated for the different datasets at different threshold detection levels. Briefly, Precision measures the number of truly detected vertices found with a given threshold level, while Recall measures the number of vertices undetected for that threshold level. The curve for the Notum dataset has an anomalous behavior for low recall values that arises from the very high values of the Vertexness function at sensory bristle cell locations that are mistaken for AJ locations. S6A Fig presents an example of this effect. Another common vertex location error that is produced at AJs are *indentations* between adjacent vertices as shown in S6B Fig. These are likely to arise by displacement of cell contacts by contractile forces generated by the actomyosin cytoskeleton. Vertex locations errors are more common in areas where cells are more parallel to the Z axis, where they are more difficult to locate due to voxel anisotropy and in areas with a low signal to noise ratio (Fig 4C and 4D).

Second, we conducted a performance assessment of the AJ edge detector. The AJ graph was initialized with the ground truth vertex locations curated for each frame in the time lapses. Fig 4E presents the Precision-Recall curves generated for the different data sets for different propagation thresholds *T*_ℰ_ of the algorithm employed to detect edges. Fig 4F shows the variations of the performance according to the value of the threshold. The method achieves high detection scores detecting vertices in the different datasets as soon as the threshold is given an appropriate value.When the threshold is too low many cells are not be detected, while when the threshold is too high extra cells that don’t exist are detected. The optimal detection results are shown in Fig 4G and 4H. Note that areas surrounding bristles and areas with low signal-to-noise ratio produce more detection errors (Fig 4G and 4H). In addition, as the orientation of edges become more parallel to the Z-axis they become more difficult to detect.

Last, we evaluated the performance of establishing cell correspondences across frames. To this end we employed all the timelapses. The method depends on 11 parameters to compute the association costs (see materials and methods for detail). As it is not practical to adjust all values for each new dataset, we searched for a combination of parameter values that works wells for the different tissues. We have exhaustively explored the parameter space and have found a combination that provides accurate tracking results across the different scenarios, with a mean *AF*1 = 0.93. This represents a very high value as the *AF*1 metric highly penalizes failures in rare events such as mitosis and apoptosis. Fig 4I, 4J, 4K and 4L present cell trajectories recovered from the different timelapses. The position over time of each cell is projected in 2D. The variations of the performance according to the perturbation of the different parameters are provided as supplementary material (S7 and S8 Figs).

To check how the sampling rate of the timelapse influences tracking performance, we repeated the search for optimal parameter values including a decimated copy of the four datasets discarding every odd frame. The performance drops to a mean *AF*1 − *score* = 0.822569. Computing the *AF*1 for the complete and decimated data set with the new parameters reveal that the *AF*1_*complete*_ = 0.92 and the *AF*1_*decimated*_ = 0.736818. This shows that the sampling rate of the data set highly influences tracking performance. Interestingly, if we compare the values found for the weights in previous and current experiments (S9 Fig), the weight given to the distance among centroids is reduced and the weight given to other features such as cell perimeter and rotation is increased.

Additionally, we compared a 2D simplification of our system to the SeedWaterSegmenter, which utilizes a watershed algorithm to detect cell extents [21], in the detection of cells, and found that our method performs better in obtaining cell locations. See supplementary material for further experimental details.

## Discussion

We have developed a computational pipeline that successfully transforms input 3D time lapse data into a rich description of cells features, connectivity and dynamics. We have shown that the pipeline detects and tracks cells with a high accuracy when properly parameterized. Clearly the most difficult part of the segmentation process is to locate the AJ vertices, as indicated by a lower F1-score, compared to the detection of edges and the computation of correspondences across frames. However, we described the errors produced by the vertex detection algorithm, so it should be straightforward to manually correct these errors to improve the accuracy of segmentation. We have also provided an example of how to use the generated cell centroid trajectories to study global cellular behaviors during tissue morphogenesis.

Our methods were developed to segment and track epithelial cells in both simple and pseudostratified epithelial monolayer such as those present in Drosophila embryo and imaginal discs and early embryos of other species including mouse and zebra fish. Thus, our methods should be generally applicable to the analysis of epithelial morphogenesis in developing embryos in a range of species. We are currently using the system to explore the morphogenesis of the epithelium of the notum and leg imaginal discs to detect patterns of cellular behavior that correlate with the regional subdivision of these structures. We are particularly interested in exploring the behaviors that contribute to the narrowing and elongation of the epithelium of the leg imaginal disc and the relative contribution of presumptive joints and segments to this process [33, 34]. In the notum, we are particularly interested in the relationship between the anterior-posterior subdivision of the notum and the pattern of cell rearrangements in each region [35, 36]. Addressing these questions will help us understand how patterning of epithelial sheets at early stages of development affect epithelial dynamics and mechanics that generate the final form of adult structures at later stages.

The proposed system can be used to investigate the topology of epithelial cells at each developmental time point, the deformation of cells and domains over time and the contributions of mitosis, apoptosis and cell movement to tissue morphogenesis. The detection of cell outlines and motion can be used to examine the evolution of cell topologies and the relative contribution of subcellular and cellular deformations to tissue morphogenesis. As the system stores the locations of vertices, edges and centroids in the data structure, it could be used to parameterize force models of epithelial remodeling such as cell vertex models [37, 38]. The detection of mitosis and apoptosis can be used to investigate the contribution of lineage relationships and patterns of apoptosis to tissue development.

A major challenge in the study of epithelial dynamics and tissue mechanics is to characterize the forces that drive planar cell rearrangements in epithelial sheets, how the forces are generated, how they affect cellular behavior [2, 22] and tissue remodeling. The activity of contractile actomyosin networks and their coupling to the AJs can alter cell shape and cell contacts, as well as spatial patterns of cell proliferation and apoptosis. In contrast, adhesive forces mediated by cell adhesion proteins promote formation and expansion of cell contacts. Differential regulation of these contractile and adhesive networks in space and time can be used to deform epithelial sheets in predictable ways. Understanding how the activities of these networks are related to the changes in cell behavior can suggest mechanisms that drive tissue morphogenesis. The proposed system might be extended to relate changes in abundance, dynamics and polarity of contractile and adhesive molecular assemblies to the changes in cell and tissue shape. The segmentation and tracking of AJs in 3D and the curation of vertices, edges, cells and trajectories would enable the extension of the pipeline to relate molecular dynamics with cell and tissue remodeling for multiscale analysis of epithelial tissue morphogenesis.

## Materials and methods

### Tissue Preparation and Image Acquisition

Pupa were collected at the white prepupal stage (0 APF) and aged in a humidified chamber at 25°C. For notum imaging, the pupal case was removed to expose the head and dorsal thorax at the desired developmental stages. For mounting, a slab of 1.5% agarose gel was placed atop a 30 mm coverslip. 2 intact pupae were mounted in tandem on the coverslip through a slit made in the agarose slab. A silicone gasket (Sylgard 184, Dow Corning) was fitted to surround the agarose slab and a chamber constructed from acrylic was fitted atop the gasket to seal the chamber [39]. For leg imaging, legs were dissected in Shields and Shang M3 insect medium and then placed in a 35-mm glass bottom microwell petri dish (P35G-1.5-14-C; MatTek) in eversion medium composed of M3 medium (S3652; Sigma), 2% fetal calf serum (10438; Gibco), 0.5% penicillin-streptomycin (15140–122; Invitrogen), 0.1 *μ*g/mL Ecdysone (20-hydroxyecdysone H5142; Sigma), 2.5% /vol methyl-cellulose (M0387-100G; Sigma) as previously described [40].

The precise settings for acquisition of time lapse movies varied for each experiment but were dictated by the need to minimize photobleaching of the E-cad∷GFP reporter, tissue damage and image corruption by noise, and to maximize image resolution (supplementary S1 Table). Approximately 10 sections at 1 to 1.5 micrometer intervals with 50% overlap were collected every 10 minutes to provide sufficient temporal resolution to track the cells and capture mitosis and apoptosis events.

The 3D volumes were deconvolved employing a custom implementation of the Richardson-Lucy algorithm [41] to remove the effects of the lens blur and the Poisson noise process introduced in the photodetector. We employ an Elastic-Net Prior [42] to perform the deconvolution, as many of the voxels are expected to be zero. CLAHE was employed to locally normalize the intensity of the 3D volumes, enhancing the AJ structures.

### Adherens Junction Segmentation

An AJ graph is built to represent the structure of the AJs in the input 3D volumes. It is an undirected graph defined by the tuple *G*_*A*_ = (*V*_*A*_, *E*_*A*_), where *V*_*A*_ is the set of AJs vertices—the points where three or more cells touch—and the set of edges *E*_*A*_ is such that there is an edge *e* ∈ *E*_*A*_ for each pair of contiguous vertices in the tissue. We build the AJ graph from the output of the plateness function 𝒫(*x*) proposed by Mosaliganti et al. [29] measuring how likely each voxel is part of the AJs and a vertexness function 𝒱(*x*) measuring how likely each voxel is an AJ vertex. Both functions are built from spatial second order derivatives of the image intensity. See supplementary material for an accurate description of the functions.

The vertex locations *V*_*A*_ = ({*v*_1_, …, *v*_*n*_} are obtained as the local maxima of the vertexness function 𝒱(*x*). A threshold value *T*_*V*_ is set up to reject spurious detections so ∀*x* ∈ *V* *T*_𝒱_ ≤ 𝒱(*x*).

**Fig 5:**
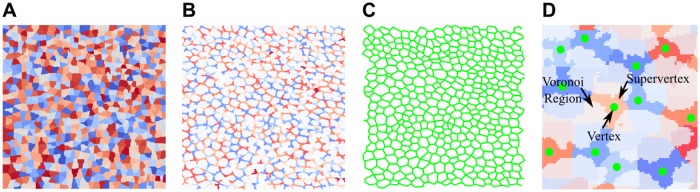
Establishing connectivity among AJ vertices to segment epithelial tissues. A) Voronoi regions are expanded from vertex locations to generate the Voronoi Diagram associating each voxel to the nearest AJ vertex. B) Supervertices are expanded inside Voronoi regions to link adjacent vertices. C) The AJs graph is built adding an edge between contiguous vertices through pairs of adjacent supervertices. D) Vertices, Voronoi regions and supervertices are superimposed.

To identify edges based on segmented vertices, we devised a method based on dividing the input space into *supervertices* (Fig 5B) and inferring their connectivity relationships. A *supervertex* is the 3D region around each vertex *v* ∈ *V*_*A*_ such that *v* is the vertex minimizing a time of travel cost function to be introduced later. We build the formal definition of a supervertex from the notion of Voronoi Region around a vertex. The Voronoi Region [43] around a vertex *v* ∈ *V*_*A*_ is the subset of all the points in the 3D space that are closer to *v* than to any other vertex in *V*_*A*_:

$$\begin{array}{c} \text{Voronoi}\;(v)=[x\in {\mathbb{R}}^{3}|\arg\min_{v^{\prime }\in V_{A}}{∥v^{\prime }-x∥}_{2}=v] \end{array}$$ (1)

The partition of the space given by the different Voronoi Regions is called a Voronoi Diagram (Fig 5A). For each point on the Voronoi Region of a vertex *v* we compute the time needed to travel to *v*, where the travel speed at each point *x* ∈ ℝ^3^ is given by *F*(*x*) = 𝒫(*x*) + 𝒱(*x*) > 0. The time needed to travel from each point *x* ∈ Voronoi(*v*) to the vertex *v* is found as the solution to the partial differential equation:

$$\begin{array}{c} F\left(x\right)|\nabla T_{v}\left(x\right)|=1 \end{array}$$ (2)

with boundary condition *T* (*v*) = 0. This equation is a well known partial differential equation known as the Eikonal equation. It is employed to obtain the travel time for a wave to reach a point when traveling through a scalar field.

Based on the notion of a Voronoi region around a vertex and the Eikonal equation employed to measure the travel-time cost from a point to its nearest vertex we provide a formal definition for a supervertex. The supervertex of a vertex *v* ∈ *V*_*A*_ is defined to be the subset of the points in the Voronoi Region of *v* such that the solution $T_{v}^{*}$ of Eq 2 with initial vertex *v* is lower than a threshold *T*_*ℰ*_:

$$\begin{array}{c} \text{Supervertex}\;(v)=[x\in \text{Voronoi}\;(v)|T_{v}\;(x)\leq T_{ℰ}] \end{array}$$ (3)

Given the set of AJ vertices *V*_*A*_ we obtain their corresponding supervertices employing a custom implementation of the Fast Marching algorithm [44] to solve Eq 2 that keeps track of the origin of the propagating wave and does not allow propagation across different Voronoi regions. An edge (*v*, *w*) is added to *E*_*A*_ if and only if supervertex(*v*) and supervertex(*w*) touch. The computed AJ graph is shown in Fig 5C.

### Cell Identification

The AJ graph computed in the previous section provides a symbolic representation of the AJ structure. However, the AJ graph does not explicitly model cells and their spatial extent and connectivity. To build a tissue model at the level of cells and edges and perform inferences, it is possible to exploit the properties of the AJ graph, and transform it into another graph to represent cells and their neighborhood relationships.

The AJ graph belongs to a class of graphs known as planar graphs [45]. Intuitively, a graph is planar when it might be drawn in a plane with none of its edges crossing one another. Formally, a graph *G* = (*V*, *E*) is planar if there exist an embedding of the vertices in ℝ^2^, *f* : *V* → ℝ^2^ such that for all pairs of edges (*a*, *b*), (*c*, *d*) ∈ *E*, with *a* ≠ *b* ≠ *c* ≠ *d* ∈ *V*, the line segment from *f*(*a*) to *f*(*b*) does not cross the line segment from *f*(*c*) to *f*(*d*). The edges of any planar graph *G* divide the space into regions called *faces*. The main implication for a graph being planar is the existence of an equivalent dual graph. For every planar graph *G* drawn in a plane there exists a graph G* whose vertices correspond to the faces of *G* and there is an edge between every pair of adjacent faces, i.e. the faces have at least one edge in common.

The planarity property of the AJ graph allows the identification of the cells that form the tissue from the stained AJs without the need to first identify cell nuclei. The Cell graph *G*_*C*_ = (*V*_*C*_, *E*_*C*_) is defined as the dual graph of *G*_*A*_, removing the vertex corresponding to the outer face. Each one of the vertices *v* ∈ *V*_*C*_ represents a cell. Each one of the edges (*v*, *w*) ∈ *E*_*C*_ represents cell adjacency among *v* and *w*. Thus, for each cell in the tissue there is a vertex in the Cell graph. A cell is defined by the pair (*c*,𝒜) where *c* ∈ *V*_*C*_ is the vertex representing the cell in the cell graph and the set *A* = *a*_1_, …, *a*_*K*_, *a*_*k*_ ∈ *V*_*A*_ is the clockwise ordered list of the vertices of the AJ graph delimiting the cell. The polygonal representation of *A*_*j*_ allows the derivation of moments such as cell perimeter *p*(*c*) ∈ ℝ^+^, cell area *a*(*c*) ∈ ℝ^+^, cell centroid *b*(*c*) ∈ ℝ^3^, cell width *w*(*c*) ∈ ℝ^+^, length *h*(*c*) ∈ ℝ^+^ or rotation *r*(*c*) ∈ (−*π*, *π*). All these features are later employed to match cells among adjacent frames for cell tracking. This representation also provides an explicit model of cell neighborhood necessary to compute tissue measurements such as those proposed in [46] or [47].

### Cell Tracking

We next developed methods to establish the correspondence among cells over time. The developed cell tracking methods rely on the methods we have developed to identify cell locations and their spatial properties. The method we employed to solve the cell correspondence problem among frames is a variation of the coupled min cost-max flow framework reported in [10]. The solution to the cell correspondence problem is obtained as the solution to a flow transportation problem in a directed graph. A total of *N* + *M* units of flow need to be sent from a source vertex *T* ^+^ to a sink vertex *T* ^−^ traversing a network formed by set of vertices and a set of arcs connecting the vertices that encodes the cell association problem. Arcs have a maximum capacity and an associated cost for sending units of flow through them. The set of arcs minimizing the cost for sending the *N* + *M* units of flow through the network gives the solution to the cell correspondence problem. Flow has to be preserved among the arcs of the network, i.e., the same amount of flow that gets into a vertex needs to be sent to others, except at source and sink vertices. The cell association cost attached to the arcs is computed from the cell moments obtained in the previous section. The cost of associating a cell to a given cell in the next frame is computed as a weighted sum of the difference among their moments. In case of a mitosis event, the cost is computed in a similar way but from the union of the polygons corresponding to a pair of adjacent hypothesized sibling cells. The weight for cells entering and leaving the scene is proportional to the distance from the centroid to the tissue perimeter. The weight for the apoptosis hypothesis is proportional to the area of the dying cell.

The exact formulation of the coupled min-cost max-flow framework employed is provided in supplementary material. The proposed framework differs from the original in the way correspondence hypotheses are formulated. As we track cells embedded in an epithelial sheet rather than freely moving particles we exploit the neighborhood relationships among cells to drop association hypothesis not corresponding to adjacent cells.

### Analysis of Notum Morphogenesis

#### Kalman filtering for trajectory postprocessing

The cell centroid trajectories extracted by our cell tracking system are not smooth, as it solves temporal correspondences among the observed positions of the cells without performing any correction to the observed positions. We employ a Kalman filter to model the joint probability distribution *P*(*O*, *H*) of the sequence of observed positions $\hat{O}={\hat{o}}_{1},{\hat{o}}_{2},\ldots ,{\hat{o}}_{T}$, ${\hat{o}}_{t}={\left({\hat{x}}_{t},{\hat{y}}_{t}\right)}^{T}\in {\mathbb{R}}^{2}$ of the centroid of a given cell and the sequence *H* = *h*_1_, *h*_2_,…,*h*_*T*_, $h_{t}={\left(x_{t},y_{t},{\dot{x}}_{t},{\dot{y}}_{t}\right)}^{T}\in {\mathbb{R}}^{4}$ of true cell state estimations [32]. The Kalman filter defines a stationary factorization of the state distribution as:

$$\begin{array}{c} P(O,H)=P\left(h_{1}\right)\prod_{t=1}^{T-1}P(h_{t+1}|h_{t})\prod_{t=1}^{T}P(o_{t}|h_{t}) \end{array}$$ (4)

where the state transition distribution *P*(*h*_*t*+1_∣*h*_*t*_) is a Gaussian distribution such *h*_*t*+1_ ∼ 𝒩(*Ah*_*t*_, Σ_*H*_) with covariance Σ_*H*_ and the observation distribution *P*(*o*_*t*_∣*h*_*t*_) is also a Gaussian distribution such *o*_*t*_ ≡ 𝒩(*Bh*_*t*_, Σ_*O*_) with covariance matrix Σ_*O*_. The state transition matrix *A* and the observation matrix *B* are respectively defined as:

$$\begin{array}{c} A=(\begin{array}{cccc} 1 & 0 & 1 & 0\\ 0 & 1 & 0 & 1\\ 0 & 0 & 1 & 0\\ 0 & 0 & 0 & 1 \end{array})\;B=(\begin{array}{cccc} 1 & 0 & 0 & 0\\ 0 & 1 & 0 & 0 \end{array}) \end{array}$$ (5)

Given an observed trajectory of cell centroids *O*, we estimate the sequence *H* of states most likely to have generated it employing the Viterbi algorithm [32]. In particular, Viterbi algorithm returns the sequence H* maximizing the posterior probability distribution *P*(*H* ∣ *O*). Using this method we obtain a smoothed estimation of the position and velocities of the cells that are employed in our next analysis.

#### Strain rate tensor for the analysis of tissue deformation

We build a strain rate tensor [31] to study the relationship among the position *p*_*i*_ = (*x*_*i*_, *y*_*i*_)^*T*^ and the velocity ${\dot{p}}_{i}={\left({\dot{x}}_{i},{\dot{y}}_{i}\right)}^{T}$ of a cell *i*. The strain rate tensor is a Jacobian matrix *J* satisfying:

$$\begin{array}{c} \underset{\dot{p}}{\underset{︸}{\left(\begin{array}{c} \dot{x}\\ \dot{y} \end{array}\right)}}=\underset{J}{\underset{︸}{\left(\begin{array}{cc} \frac{\partial \dot{x}}{\partial x} & \frac{\partial \dot{y}}{\partial x}\\ \frac{\partial \dot{x}}{\partial y} & \frac{\partial \dot{y}}{\partial y} \end{array}\right)}}\underset{p}{\underset{︸}{\left(\begin{array}{c} x\\ y \end{array}\right)}}+\underset{{\dot{p}}_{0}}{\underset{︸}{\left(\begin{array}{c} {\dot{x}}_{0}\\ {\dot{y}}_{0} \end{array}\right)}} \end{array}$$ (6)

In practice, the value of *J* and the intercepts ${\dot{p}}_{0}$ are estimated from the data employing linear regression. The strain rate tensor might be decomposed into a symmetric part $ℰ=\frac{1}{2}(J+J^{T})$ and an antisymmetric part $\Omega =\frac{1}{2}(J-J^{T})$. The symmetric part provides the expansion coefficient of the vector field as trace(ℰ), while the antisymmetric part provides the rotation coefficient as acos(Ω_12_). The temporal evolution of these values provides a quantitative description of the global behavior of the tissue.

### Performance Evaluation

To assess the performance of the proposed algorithms to respectively detect AJ vertices, AJ edges and compute cell correspondences among frames we neeed to define a measure of the quality of a given configuration. There are two type of errors that detection algorithms might produce, known as False Positives (FP) and False Negatives (FN). A FP is produced when the algorithm marks a detection that is not real, while a FN is produced when the algorithm misses a real detection. Thus, to assess the quality of a given set of detections, it is possible to compute three metrics known as Precision, Recall and F1 measure defined as follows:

$$\begin{array}{c} \;\text{Precision}\;=\frac{\left|\text{Marked}\;\text{detections}\cap \;\text{Real}\;\text{detections}\right|}{\left|\text{Marked}\;\text{Detections}\right|} \end{array}$$ (7)

$$\begin{array}{c} \;\text{Recall}\;=\frac{\left|\text{Marked}\;\text{detections}\cap \;\text{Real}\;\text{detections}\right|}{\left|\text{Real}\;\text{Detections}\right|} \end{array}$$ (8)

$$\begin{array}{c} \;\text{F1}\;\text{Measure}\;=\frac{2\times \text{Precision}\times \text{Recall}}{\text{Precision}\;+\;\text{Recall}} \end{array}$$ (9)

Precision measures the amount of FP errors produced, Recall measures the number of FN the detector produces, while the F1 measure is the harmonic mean of both measures and might be understood as a summary of them. Detectors commonly have a detection threshold (the proposed here do) that has to be adjusted *a priori* to some value and conditions Precision and Recall values of the algorithm. Note that Precision and Recall are conflicting measures: a high detection threshold produces high precision and low recall (very few but true detections, but many missed detections), while a low detection threshold produces low precision but a high recall (not a lot of missed detections, but a lot of false detections). Thus, algorithms assessment should include tests for many threshold levels to produce Precision-Recall curves. The sensitivity of parameters was explored based on the changes of the F1 measures.

Similar metrics are employed to evaluate cell tracking algorithm. To this end we consider it as a label prediction problem. For each cell in a given frame the task is to predict the correspondence of a cell with the same cell in next frame, if it should disappear following apoptosis, or if it should be associated with two new cells following mitosis. Thus, an average *F*1 measure might be obtained for each tracking configuration as the harmonic mean of the individual *F*1 measures:

$$\begin{array}{c} AF1=\frac{4}{F1_{association}^{-1}+F1_{creation}^{-1}+F1_{termination}^{-1}+F1_{mitosis}^{-1}} \end{array}$$ (10)

## Supporting Information

S1 FigAdherens Junction detection at multiple spatial scales.The output of the plateness function proposed by Mosaliganti et al. is employed to detect the AJs [29]. A-C) Slices of the outupt of the plateness function 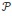
_*σ*_(**x**) computed for different values of *σ* (A *σ* = 0.14, B *σ* = 0.45 and C *σ* = 0.60). The value of 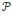
_*σ*_ (**x**) depends on the signal intensity variations among AJs produced by the imaging process and variations in the structure of AJs, achieving the maxima at real scale *σ*.(TIFF)

S2 Fig Membrane enhancement diffusion improves detectability of AJs [29]. A) A slice of the planarity response at *σ* = 0.14 before applying diffusion. Planarity values are noisy and the response at the AJs is uneven. B) The same slice after 50 iterations of membrane enhancement diffusion. Most of the noisy values have been removed, the detected AJs obtain a more uniform intensity profile, and some of the cytoplasmic signal has been cleared.
(TIFF)

S3 FigThe output of the plateness function proposed by Mosaliganti et al. [29] decreases around AJs vertices.Arrows point to voxels where the effect is well observed. This feature is exploited to detect cell vertices. The image shows a slice of the ouput of the plateness function 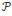
_*σ*_(**x**) with *σ* = 0.6.(TIFF)

S4 Fig AJs Vertex location. The output of the Vertexness function here proposed has been overimposed in black over the plateness function outputs shown in S1 Fig at the corresponding scales (A *σ* = 0.14, B *σ* = 0.45 and C *σ* = 0.60). Note that at higher scales (B and C) vertices which are close to each other tend to merge, while at lower scales vertices tend to appear at non-vertex locations along the AJs. A set up such as the one proposed in panel B is desired as it provides an accurate detection of AJs and vertices. In A the scale is too low resulting in high noise, while in C the scale is too high resulting in detection of blurred features.
(TIFF)

S5 Fig Solution to the correspondence among the cells in a hypothetical epithelial tissue. A) Two cells, l2 divides to produces r2 and r3. B) The graph we built to represent all the correspondence hypotheses. Arcs in red represent cell association, in blue cells entering the scene, in green mitosis, in pink apoptosis and in gray cells leaving the scene. C) The arcs of the graph expected to represent the desired solution
(TIFF)

S6 Fig Typical errors of vertex detection. Details from Fig 4C. Green vertices represent true detections, blue, missed detections, and red, false detections. A) Common pattern of vertices detected at bristle locations, where many vertices are not detected but one is falsely detected at the center. B) *Indentations* appear along edges between vertices as regions with high curvature that are detected as vertices.
(TIFF)

S7 Fig Variation of the tracking performance according to the weights given to the distances between the different features. Global shows the harmonic mean of the Average F1-scores obtained for the different datasets. The difference at the optimal between the global measure and the Average F1-scores of each dataset is not significant, but the global measure drops fast as parameter values deviate from the optimal. A) Centroids. B) Area. C) Perimeter. D) Width. E) Rotation. F) Length.
(TIFF)

S8 Fig Variation of the tracking performance according to the weights given to the different hypotheses. Similar to S7 Fig, global shows the harmonic mean of the Average F1-scores obtained for the different datasets. The difference at the optimal between the global measure and the Average F1-scores of each dataset is not significant, but the global measure drops fast as parameter values deviate from the optimal. A) Cell Association. B) Cell entering the scene. C) Cell mitosis. D) Cell Apoptosis. E) Cell leaving the scene.
(TIFF)

S9 Fig Values of the optimal weights given to the distance among the different cell features employed to compute cell association hypotheses to track cells. *w*_*b*_, *w*_*a*_, *w*_*p*_, *w*_*w*_, *w*_*θ*_ and *w*_*h*_ are respectively the weights given to the the distances among cell centroids, areas, perimeters, widths, rotations and heights to compute cell association costs. The distance between cell centroids (*w*_*b*_) receives less importance in tracking cells in decimated data, while the distance between cells perimeters (*w*_*p*_) and rotation (*w*_*θ*_) receives more importance.
(TIFF)

S10 Fig Comparison between the performance of TTT (simplified to segment the AJs in 2D) and SeedWaterSegmenter segmenting the cells in the Notum dataset. TTT obtains a F1 score higher than SeedWaterSegmenter. These F1 scores have been obtained after tuning the parameters of SeedWaterSegmenter and TTT to obtain the highest score for each system. For SeedWaterSegmenter only one parameter was tuned, compared to seven parameters that were tuned for TTT. Although SeedWaterSegmenter is less accurate than TTT, it is easier to use.
(TIFF)

S11 Fig A screenshot of the validation tools in TTT. Vertices and edges of the AJ graph can be edited to correct for segmentation errors and obtain accurate data for further analysis. Vertices and edges are selected employing the mouse. A 3D cursor is employed to remove, add or move vertices. Note the red vertex selected by the 3D cursor.
(TIFF)

S1 Text Additional material describing technical details of the proposed system. (PDF)

S1 Table Description of the datasets employed to evaluate the segmentation and tracking pipeline. (TIFF)
