# Supplementary figures and images for: Segmentation and Tracking of Adherens Junctions in 3D for the Analysis of Epithelial Tissue Morphogenesis
Source: PLoS Comput Biol. 2015 Apr 17;11(4):e1004124. doi: 10.1371/journal.pcbi.1004124 (PMC4401792; doi:10.1371/journal.pcbi.1004124)

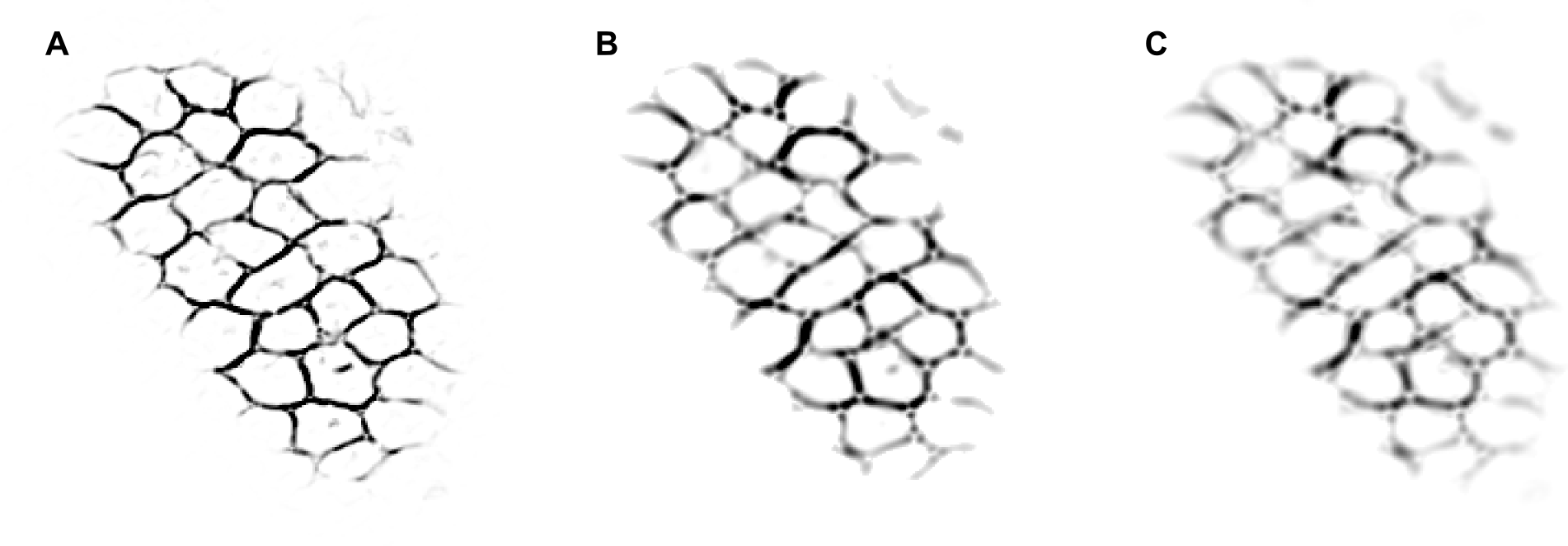

Supplement: S1 Fig — The output of the plateness function proposed by Mosaliganti et al. is employed to detect the AJs [29]. A-C) Slices of the outupt of the plateness function σ(x) computed for different values of σ (A σ = 0.14, B σ = 0.45 and C σ = 0.60). The value of σ (x) depends on the signal intensity variations among AJs produced by the imaging process and variations in the structure of AJs, achieving the maxima at real scale σ. (TIFF) [file pcbi.1004124.s001.tiff]

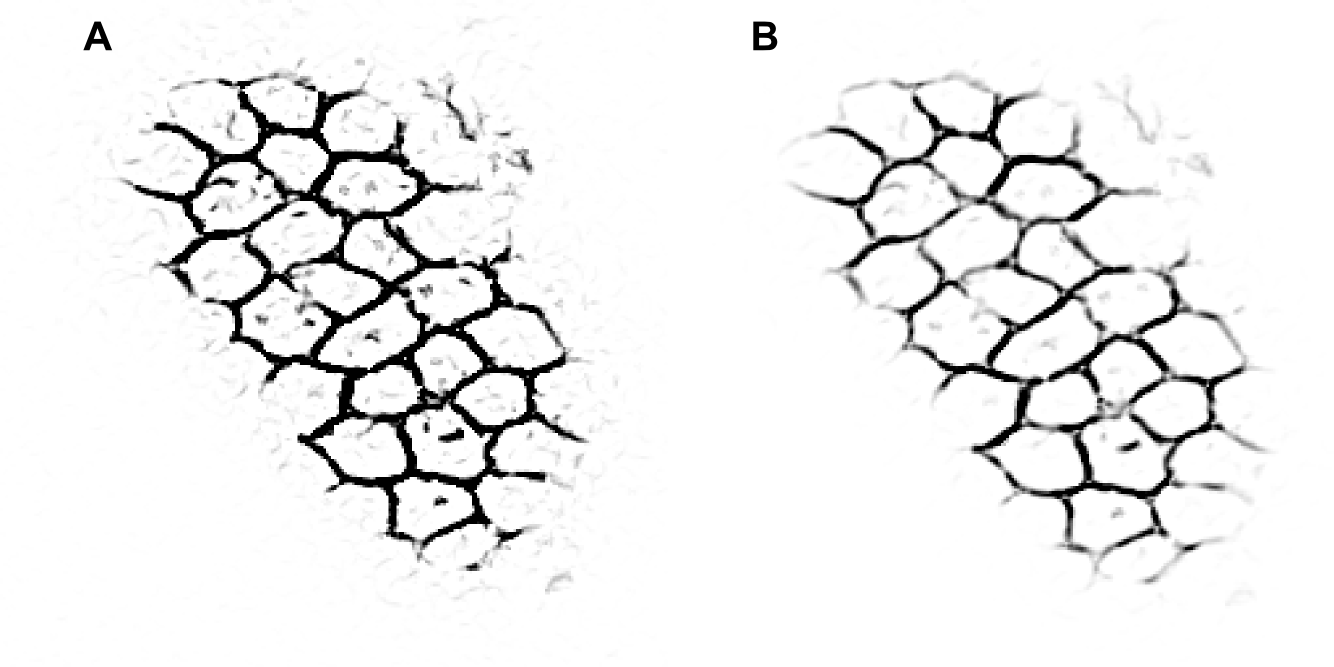

Supplement: S2 Fig — A) A slice of the planarity response at σ = 0.14 before applying diffusion. Planarity values are noisy and the response at the AJs is uneven. B) The same slice after 50 iterations of membrane enhancement diffusion. Most of the noisy values have been removed, the detected AJs obtain a more uniform intensity profile, and some of the cytoplasmic signal has been cleared. (TIFF) [file pcbi.1004124.s002.tiff]

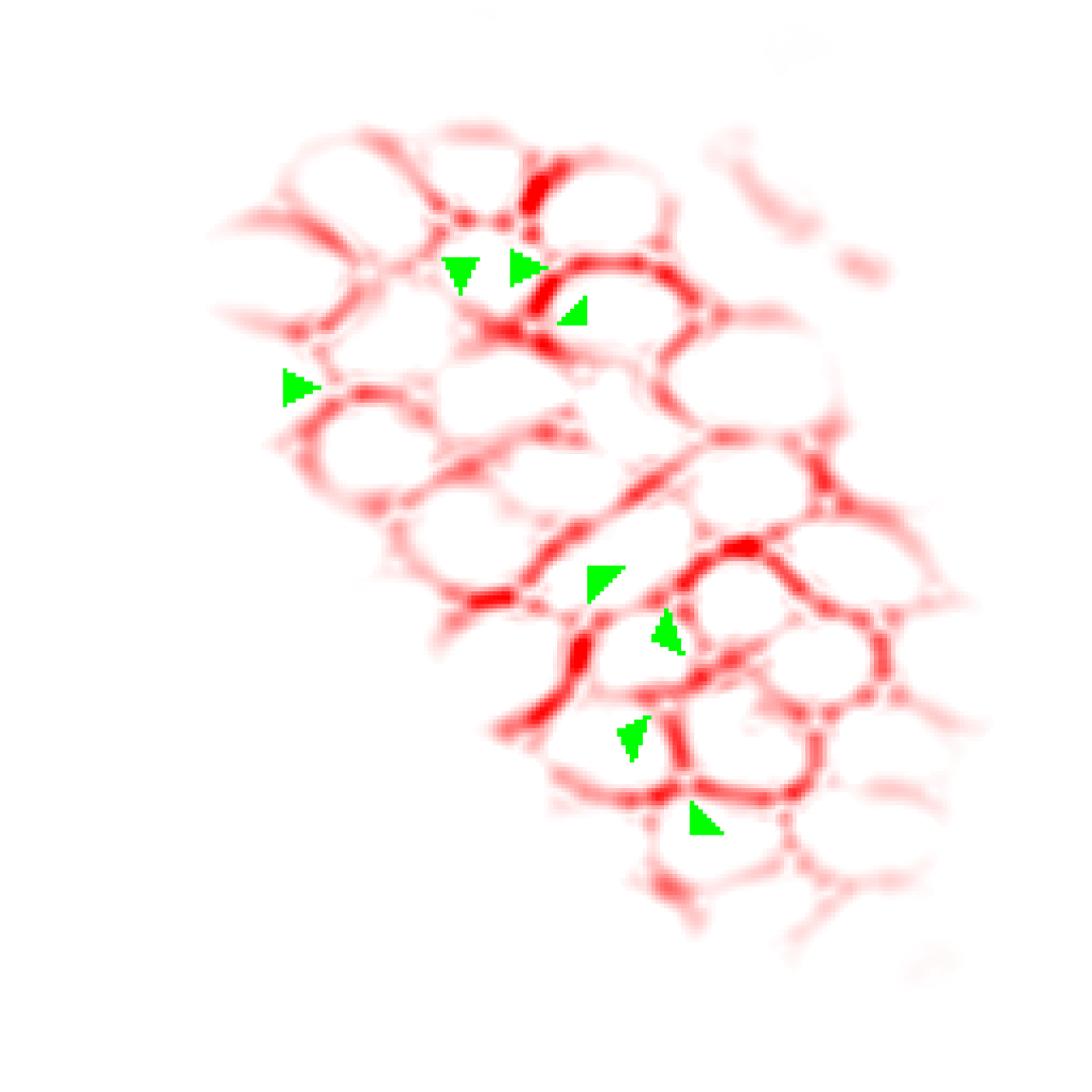

Supplement: S3 Fig — Arrows point to voxels where the effect is well observed. This feature is exploited to detect cell vertices. The image shows a slice of the ouput of the plateness function σ(x) with σ = 0.6. (TIFF) [file pcbi.1004124.s003.tiff]

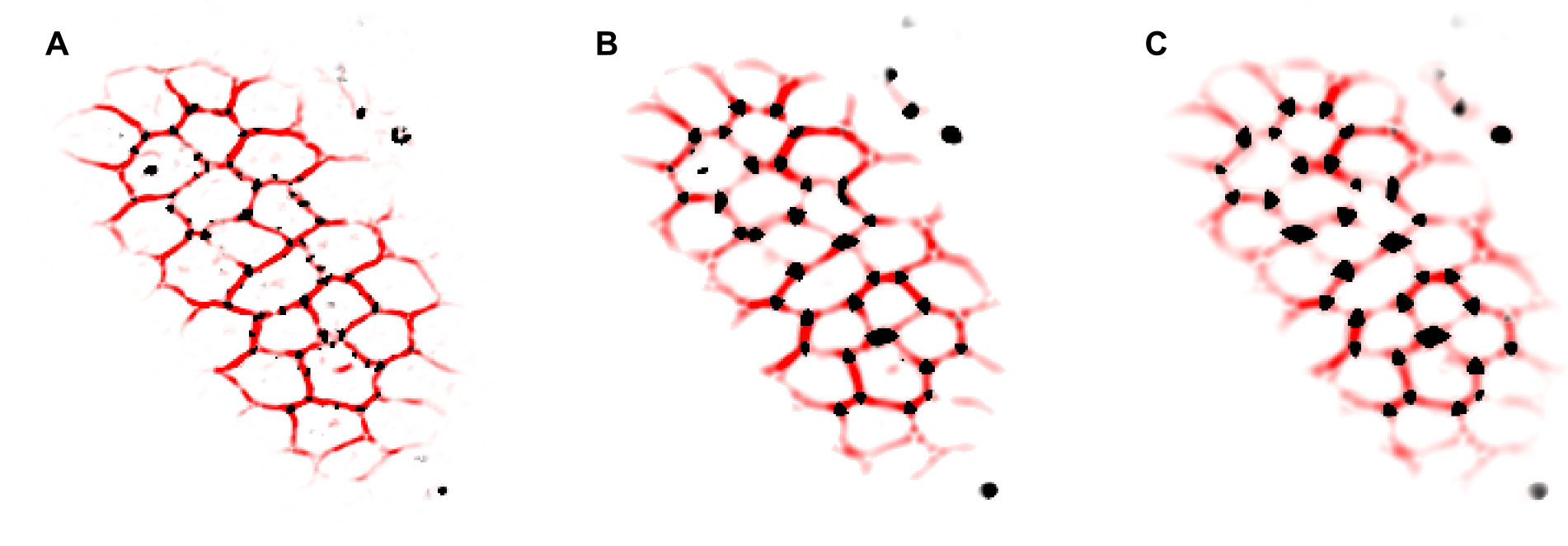

Supplement: S4 Fig — The output of the Vertexness function here proposed has been overimposed in black over the plateness function outputs shown in S1 Fig at the corresponding scales (A σ = 0.14, B σ = 0.45 and C σ = 0.60). Note that at higher scales (B and C) vertices which are close to each other tend to merge, while at lower scales vertices tend to appear at non-vertex locations along the AJs. A set up such as the one proposed in panel B is desired as it provides an accurate detection of AJs and vertices. In A the scale is too low resulting in high noise, while in C the scale is too high resulting in detection of blurred features. (TIFF) [file pcbi.1004124.s004.tiff]

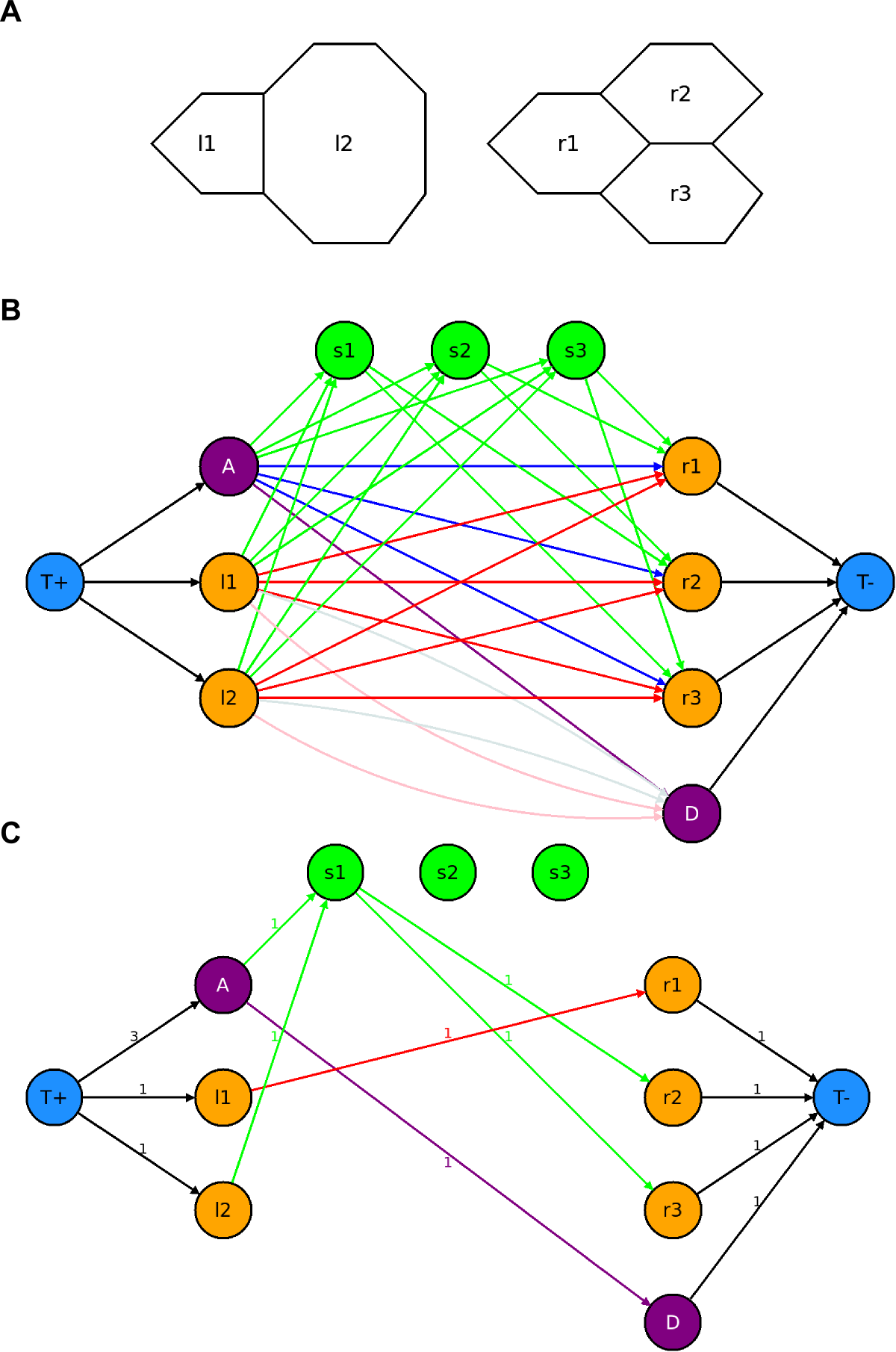

Supplement: S5 Fig — A) Two cells, l2 divides to produces r2 and r3. B) The graph we built to represent all the correspondence hypotheses. Arcs in red represent cell association, in blue cells entering the scene, in green mitosis, in pink apoptosis and in gray cells leaving the scene. C) The arcs of the graph expected to represent the desired solution (TIFF) [file pcbi.1004124.s005.tiff]

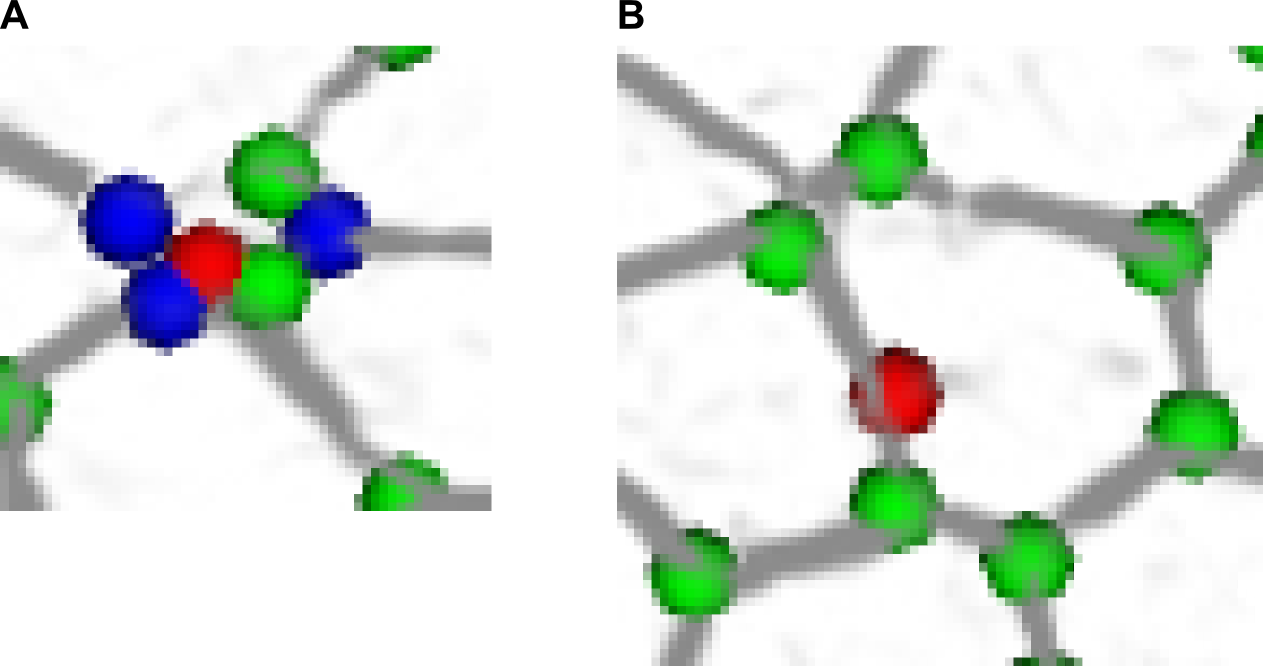

Supplement: S6 Fig — Details from Fig 4C. Green vertices represent true detections, blue, missed detections, and red, false detections. A) Common pattern of vertices detected at bristle locations, where many vertices are not detected but one is falsely detected at the center. B) Indentations appear along edges between vertices as regions with high curvature that are detected as vertices. (TIFF) [file pcbi.1004124.s006.tiff]

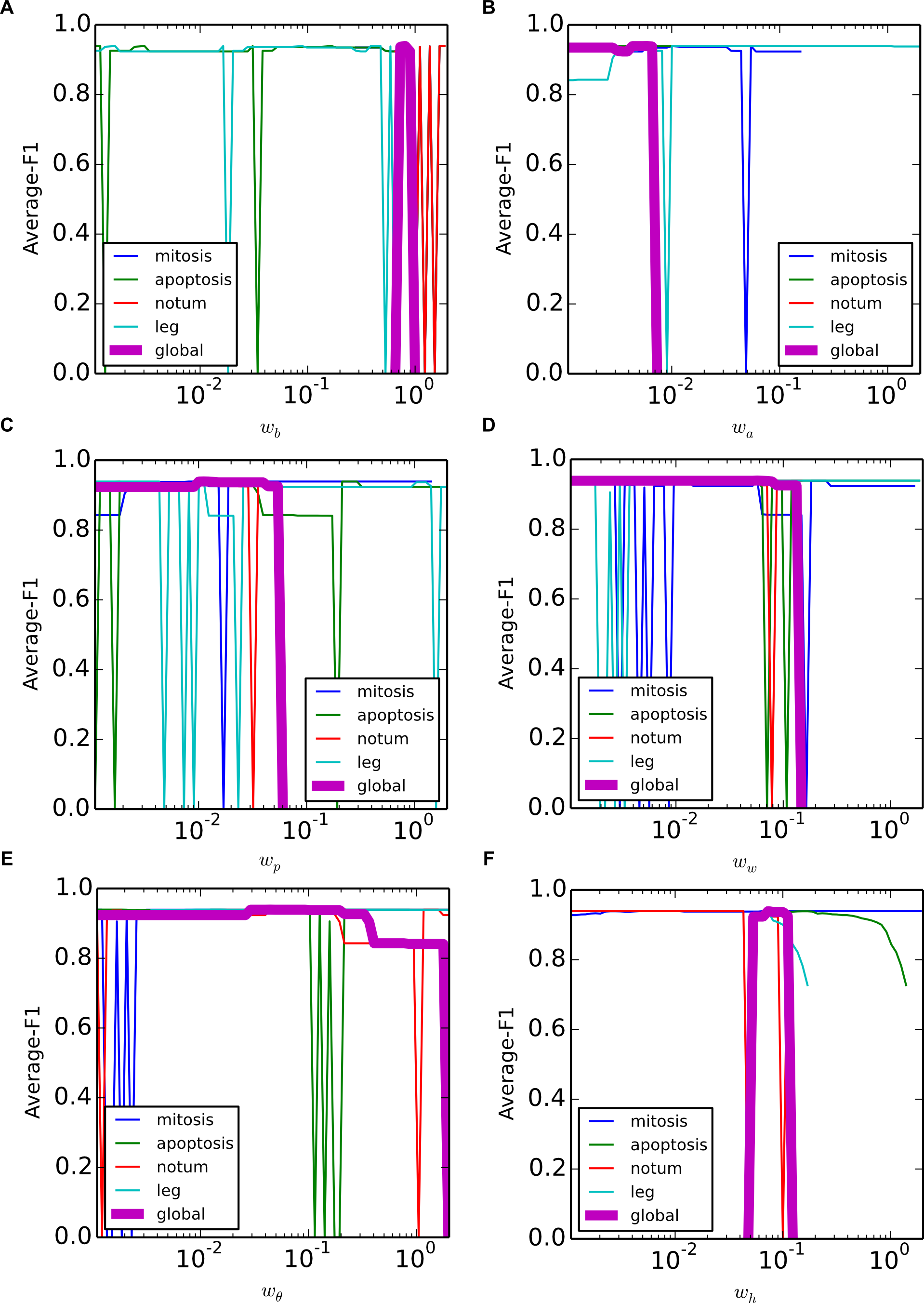

Supplement: S7 Fig — Global shows the harmonic mean of the Average F1-scores obtained for the different datasets. The difference at the optimal between the global measure and the Average F1-scores of each dataset is not significant, but the global measure drops fast as parameter values deviate from the optimal. A) Centroids. B) Area. C) Perimeter. D) Width. E) Rotation. F) Length. (TIFF) [file pcbi.1004124.s007.tiff]

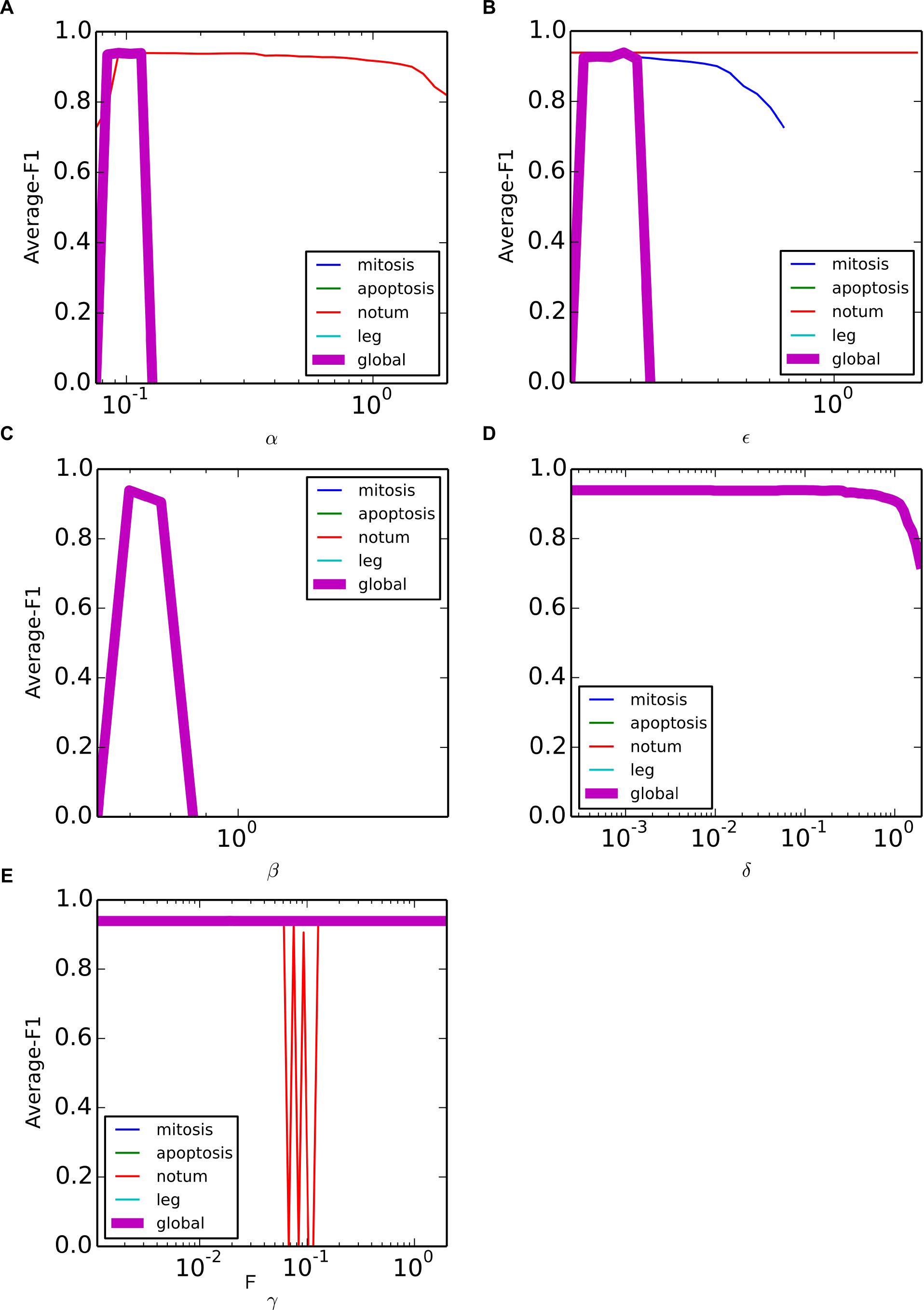

Supplement: S8 Fig — Similar to S7 Fig, global shows the harmonic mean of the Average F1-scores obtained for the different datasets. The difference at the optimal between the global measure and the Average F1-scores of each dataset is not significant, but the global measure drops fast as parameter values deviate from the optimal. A) Cell Association. B) Cell entering the scene. C) Cell mitosis. D) Cell Apoptosis. E) Cell leaving the scene. (TIFF) [file pcbi.1004124.s008.tiff]

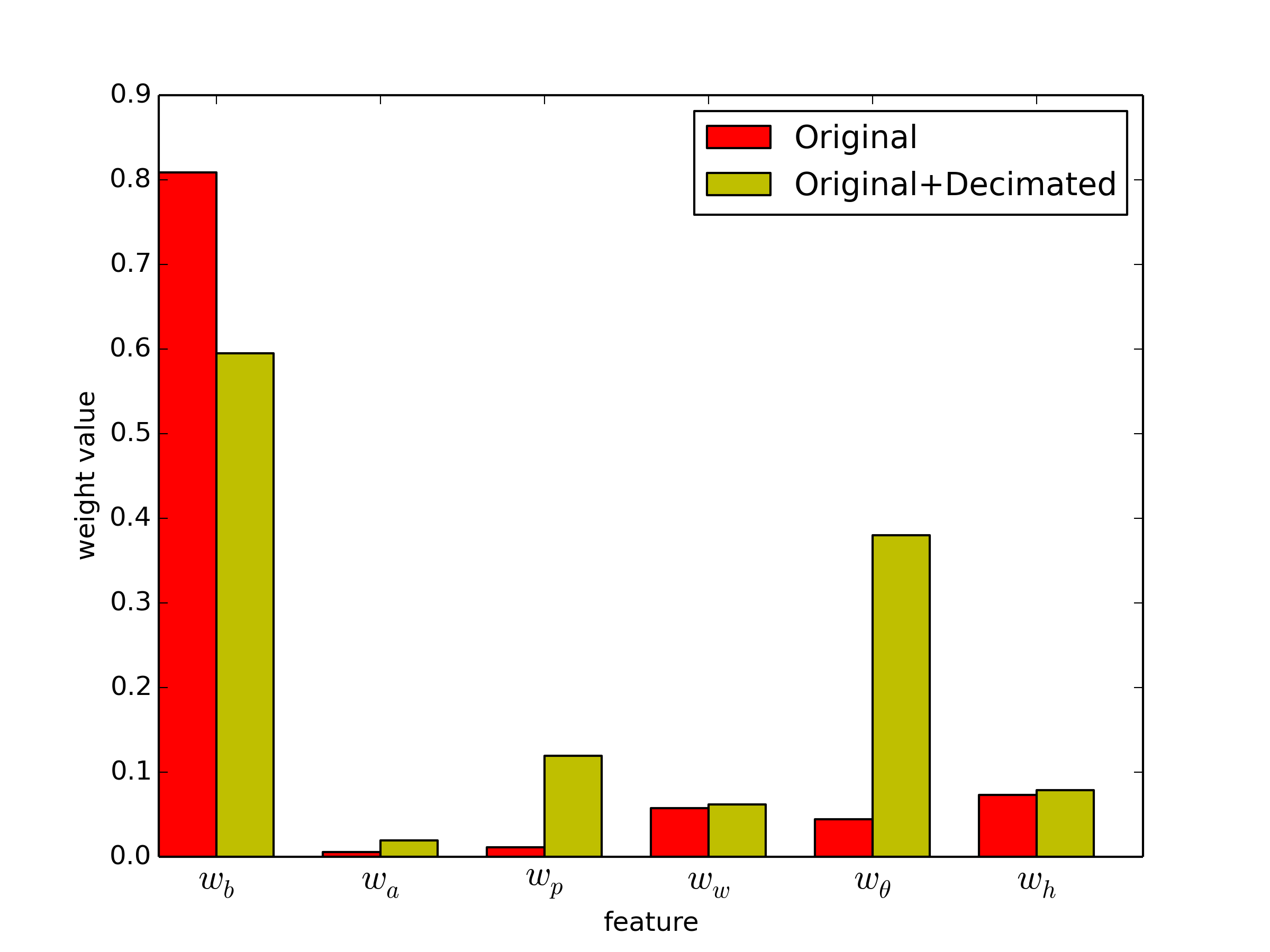

Supplement: S9 Fig — w b, w a, w p, w w, w θ and w h are respectively the weights given to the the distances among cell centroids, areas, perimeters, widths, rotations and heights to compute cell association costs. The distance between cell centroids (w b) receives less importance in tracking cells in decimated data, while the distance between cells perimeters (w p) and rotation (w θ) receives more importance. (TIFF) [file pcbi.1004124.s009.tiff]

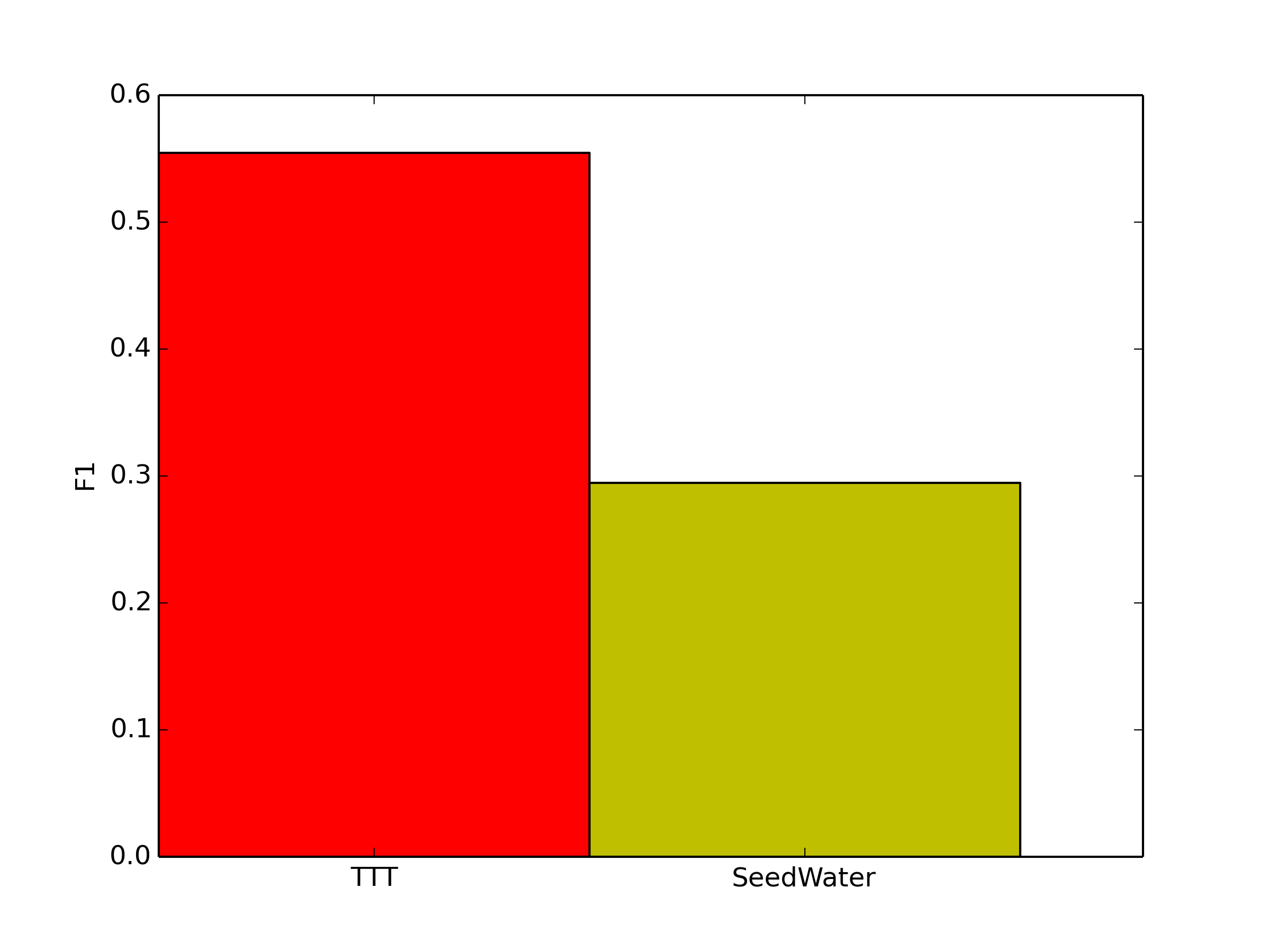

Supplement: S10 Fig — TTT obtains a F1 score higher than SeedWaterSegmenter. These F1 scores have been obtained after tuning the parameters of SeedWaterSegmenter and TTT to obtain the highest score for each system. For SeedWaterSegmenter only one parameter was tuned, compared to seven parameters that were tuned for TTT. Although SeedWaterSegmenter is less accurate than TTT, it is easier to use. (TIFF) [file pcbi.1004124.s010.tiff]

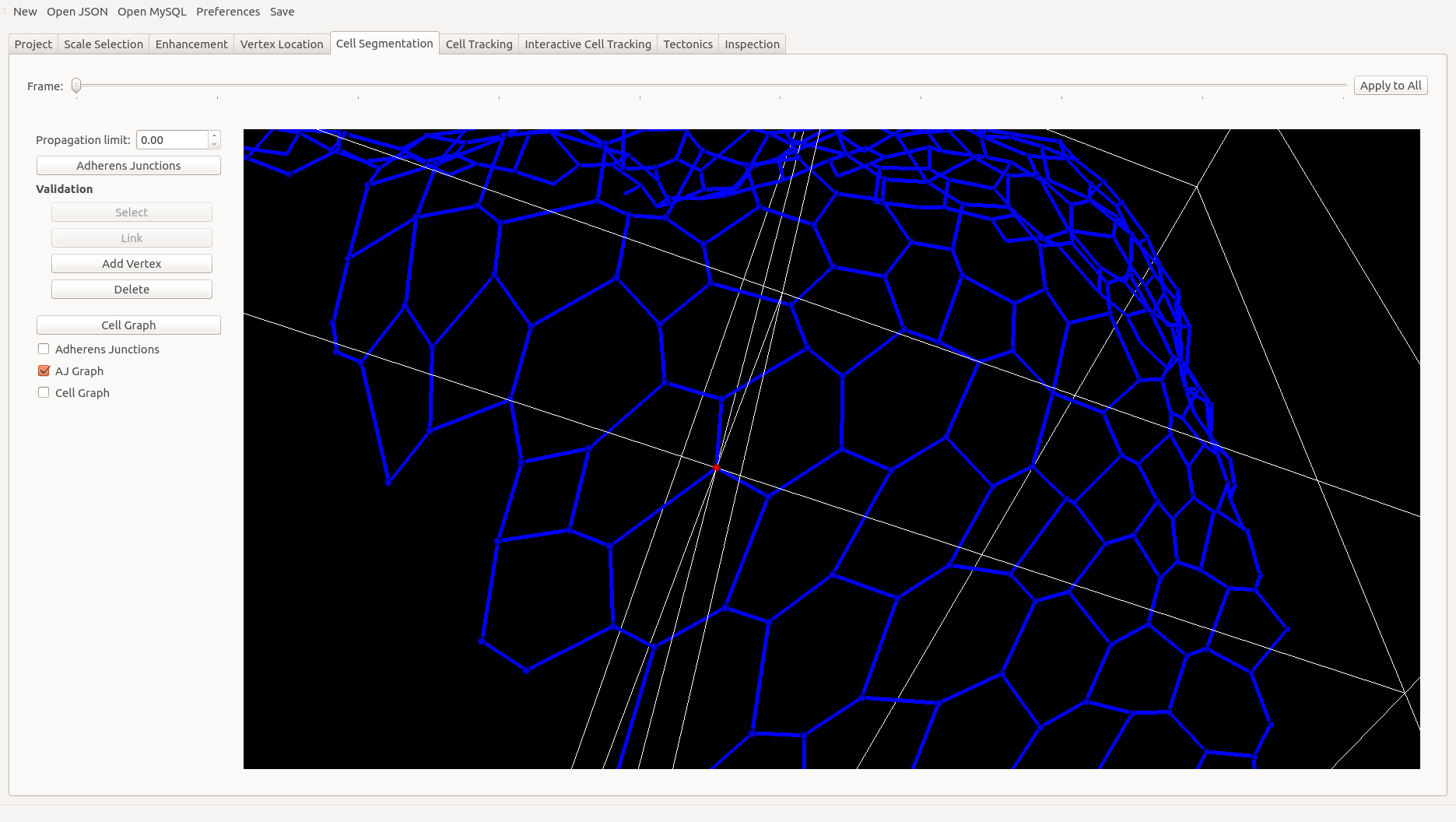

Supplement: S11 Fig — Vertices and edges of the AJ graph can be edited to correct for segmentation errors and obtain accurate data for further analysis. Vertices and edges are selected employing the mouse. A 3D cursor is employed to remove, add or move vertices. Note the red vertex selected by the 3D cursor. (TIFF) [file pcbi.1004124.s011.tiff]

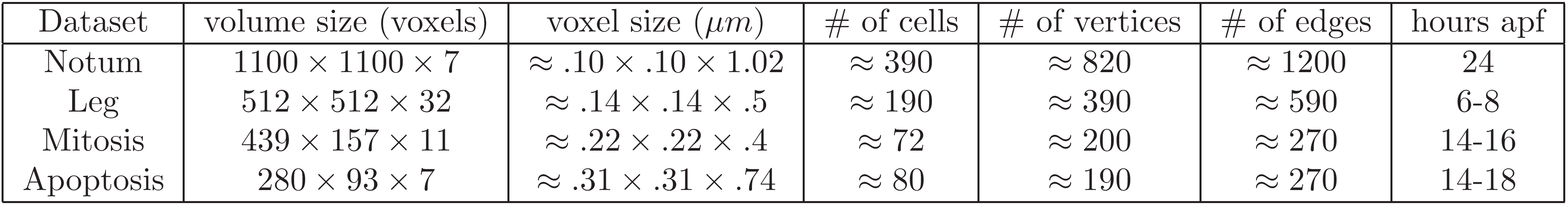

Supplement: S1 Table — (TIFF) [file pcbi.1004124.s013.tiff]
