# Supplementary material for: Segmentation and Tracking of Adherens Junctions in 3D for the Analysis of Epithelial Tissue Morphogenesis
Source: PLoS Comput Biol. 2015 Apr 17;11(4):e1004124. doi: 10.1371/journal.pcbi.1004124 (PMC4401792; doi:10.1371/journal.pcbi.1004124)
Supplement: S1 Text — (PDF) [file pcbi.1004124.s012.pdf]

Supplementary Material

Extended materials and methods

Adherens Junction Location

The AJs localize below the apical surface of the epithelium and form a belt-like structure orthogonal to that surface. Mosaliganti et al. have recently introduced a function to measure the likelihood of voxels being part of bright planar like structures, and employed it to detect the entire cell surface of somitic cells in zebrafish embryos [1]. We found that this function can detect the AJs in 3D space as a planar-like part of the cell surface, and therefore employed it to detect AJs (Fig S1).

Intuitively, the planarity function exploits the fact that the image intensity values in the neighborhood of a voxel in a planar-like structure changes slowly along some planes in the 3D space. By contrast, these values change very rapidly in the direction normal to these planes. Thus, the planarity function locates the plane of minimum variation around each voxel and builds a measure of the relative change of signal intensity between the plane and the direction normal to the plane.

The plane of minimum intensity variation around a voxel x is computed from the eigendecomposition of the Hessian Matrix $\nabla^2 u$ of the image intensity function $\mathcal{U}(\mathbf{x})$. Gaussian derivatives are employed to obtain a spatially regularized Hessian matrix $\nabla^2_\sigma U$, incorporating a local scale parameter $\sigma > 0$ to control the size of the neighborhood employed in the regularization. Be $|\lambda_1| \leq |\lambda_2| \leq |\lambda_3|$ the eigenvalues of $\nabla^2_\sigma u$ with corresponding eigenvalues e_1, e_2 e_3. The plane of minimum variation around voxel x is spanned by eigenvectors e_1 and e_2, expecting $\lambda_3 \ll \lambda_2 \approx \lambda_1 < 0$. The planarity function defined by Mosaliganti et al. to measure this property is given by:

$$\mathcal{P}_\sigma(\mathbf{x}) = \begin{cases} 0 & \text{if } \lambda_3 < 0 \\ \left(1 - e^{\frac{-S^2}{2\gamma^2}}\right) e^{\frac{-A^2}{2\alpha^2}} e^{\frac{-B^2}{2\beta^2}} e^{\frac{-2c^2}{\lambda_3^2}} & \mathit{othersise} \end{cases} \tag{1}$$

where $A = \frac{|\lambda_2|}{|\lambda_3|}$, $B = \frac{\sqrt{|\lambda_1 \lambda_2|}}{|\lambda_3|}$, $S = \sqrt{\lambda_1^2 + \lambda_2^2 + \lambda_3^2}$ and α,β,γ and c are some positive constants. However, not every voxel x has the same characteristic scale σ and the planarity value varies according to it, depending on the spatial extent of the structure. Figs 6A, 6B and 6C present planarity measurements at different scales. The planarity value of a voxel to be employed in subsequent segmentation steps is selected as the maximum value across a range of scales to account for signal intensity variations among AJs produced by the imaging process and the variations in the structure of the AJs:

$$\mathcal{P}(\mathbf{x}) = \max_{\sigma_{min} \leq \sigma \leq \sigma_{max}} \mathcal{P}_\sigma(\mathbf{x}) \tag{2}$$

for a set of scale values in the interval $[\sigma_{min}, \sigma_{max}]$.

The volume of the AJs detected by the planarity function presents defects due to the uneven distribution of E-cad at the cell membrane, residual noise and non-junctional signal in transport vesicles in the cytoplasm. A membraness enhancement diffusion scheme also proposed by Mosaliganti et al. [1] enhances the planarity properties of the plasma membrane simulating an anisotropic heat diffusion process where the maximum propagation is done among the minimum variation plane of each voxel. Employing this scheme serves to enhance the planarity properties of the adherens junction volumes $\mathcal{P}(\mathbf{x})$, fill gaps in the adherens junctions and reduce the planarity response of fluorescent signal in the cytoplasm. The effects of this process might be seen in Fig S2. The details of the method are out of the scope of this paper. Readers are referred to the original publication for additional details.

Adherens Junction Vertex Location

One approach to describe symbolically networks of epithelial cells is to identify vertices where three or more cells meet and edges between adjacent pairs of cells. Our system detects vertices and employs them as the input for the cell segmentation algorithm proposed in the next section. To this end we define a function similar to the one used in the previous section to locate AJs.

A visual inspection of the planarity function slices shown in Fig S1 reveals that the planarity measurement decreases at adherens junction vertices, as shown in Fig S3. In these regions image intensity is no longer constant among some planes but instead changes in many directions, and appears more similar to a blob than to plate. This fact is reflected by the Hessian eigenvalue magnitudes, as λ_1 and λ_2 increase their magnitude and become similar to λ_3. Now the relationship among Hessian eigenvalue magnitudes that holds is $0 \ll |\lambda_1| \approx |\lambda_2| \approx |\lambda_3|$. Thus, similar to the plateness function, a vertexness function is defined to measure how likely a voxel x is a vertex:

$$\mathcal{V}_\sigma(\mathbf{x}) = \begin{cases} 0 & \text{if } \lambda_3 < 0 \\ \left(1 - e^{\frac{-S^2}{2\gamma^2}}\right)\left(1 - e^{\frac{-A^2}{2\alpha^2}}\right)\left(1 - e^{\frac{-B^2}{2\beta^2}}\right) e^{\frac{-2c^2}{\lambda_3^2}} & otherwise \end{cases} \tag{3}$$

where now $A = \frac{|\lambda_2|}{|\lambda_3|}$, $B = \frac{|\lambda_1|}{|\lambda_2|}$ and again $S = \sqrt{\lambda_1^2 + \lambda_2^2 + \lambda_3^2}$. Again, not every voxel has the same scale property σ (see Fig S4) the vertexness value varies according to it. Thus, again, the vertexness of a voxel is selected as the maximum across a set of scales:

$$\mathcal{V}(\mathbf{x}) = \max_{\sigma_{min} \leq \sigma \leq \sigma_{max}} \mathcal{V}_\sigma(x) \tag{4}$$

between a range of values σ_{min} σ_{max}.

The vertex locations $V = \{v_1, ..., v_n\}$ are obtained as the local maxima of $\mathcal{V}(x)$. A threshold value T_V is set up to reject spurious detections so $\forall x \in V \quad T_\mathcal{V} \leq \mathcal{V}(x)$.

Cell tracking

Be $C^t = [c_1^t \dots c_N^t]$ and $C^{t+1} = \left[c_1^{t+1} \dots c_M^{t+1}\right]$ sets of N and M cells respectively extracted at adjacent sampling times t and $t+1$. The temporal correspondence problem to solve is to establish the relationship among the cells in C^t and C^{t+1}, that is, determine which cells in C^{t+1} are the result of a morphing operation over a cell in C^t, which pairs of cells in C^{t+1} are the product of the mitosis of cells in C^t, which of the elements in C^{t+1} are new cells that have entered the scene and thus not have a corresponding cell in C^t, which of the cells in C^t have disappeared at $t+1$ by an apoptosis event so do not have any correspondence in C^{t+1} and which of the cells in C^t have left the scene so have no correspondence in C^{t+1}.

The method we propose to solve the cell correspondence problem among frames is a variation of the coupled min cost-max flow framework proposed in [2]. The solution to the cell correspondence problem is obtained as the solution to a flow transportation problem in a directed graph. A total of $N+M$ units of flow need to be sent from a source vertex T^+ to a sink vertex T^- traversing a network formed by set of vertices and a set of arcs connecting the vertices that encodes the cell association problem. Arcs have a maximum capacity and an associated cost for sending units of flow through them. The set of arcs minimizing the cost for sending the $N+M$ units of flow through the network gives the solution to the cell correspondence problem. Flow has to be preserved among the network, i.e., the same amount of flow that gets into a vertex needs to be sent to others, except at source and sink vertices.

The coupled min-cost max-flow framework we employ to solve the cell correspondence problem differs from the original in the way correspondence hypotheses are formulated. As we track epithelial cells in a tissue rather than freely moving particles, we exploit certain neighborhood relationships among the cells that allow us to not consider association hypotheses corresponding to distant cells. We have found that

only considering the association hypothesis per cell for the K=3 nearest neighbors is sufficient to solve the cell correspondence problem, thus reducing the computational complexity attached to the association problem. Below we restrict our exposition to the procedure followed to formulate cell correspondence hypotheses and build the flow transport graph. Readers interested in additional details about the coupled min-cost max-flow framework are referred to the original publication [2].

The directed graph employed to model the flow transportation problem (Fig S5) is the graph $G = (V, E)$ with vertices given by $V = T^+ \cup A \cup L \cup S \cup R \cup T^-$ and arcs E. T^+ is the source vertex sending $N + M$ units of flow to the network. T^- is the sink vertex, receiving $N + M$ units of flow from the network. $L \equiv C^t$ are the vertices representing the cells at instant t. $R \equiv C^{t+1}$ are the vertices representing the cells at instant $t + 1$. A is a vertex employed to represent new cells entering the scene at $t + 1$. D is a vertex employed to represent the destruction of cells at t either by apoptosis or by leaving the scene the scene. S are auxiliary vertices employed to formulate cell mitosis hypotheses, with a vertex in S for each one of the mitosis hypothesis.

First we add to the network a set of arcs employed to route flow from the source and the sink nodes to other vertices in the network that truly model cell correspondence hypotheses. In this way an arc is added from the source vertex T^+ to each one of the vertices $l_i \in L$ representing cells from instant t, setting capacity$(T^+, l_i) = 1$ and $cost(T^+, l_i) = 0$. Similarly, an arc is added from each vertex $r_i \in R$ representing the cells at instant $t + 1$ to the sink vertex T^-, setting capacity$(r_i, T^-) = 1$ and cost$(r_i, T^-) = 0$. Arcs are also added from T^+ to A, from A to D and from D to T^- setting capacity$(T^+, A) = |L| + |R|$ and cost$(T^+, A) = 0$, capacity$(A, D) = |L| + |R|$, cost$(A, D) = 0$, capacity$(D, T^-) = |L| + |R|$ and cost$(D, T^-) = 0$.

Now that all those auxiliary arcs have been added to the flow transportation graph we start formulating cell correspondence hypotheses adding the necessary arcs to represent them. We begin formulating hypotheses about cells at $t + 1$ being the result of some morphing operation over cells at t. An arc in the flow graph from a vertex $l \in L$ to a vertex $r \in R$ represents the hypothesis that the cell r in C^{t+1} is the result of some morphing operation over the cell $l \in C^t$. For each cell $r_i \in C^{t+1}$ we formulate a set of $K = 50$ morphing hypotheses each one respectively postulating r_i as the product of a morphing operation over each cell l_j in the set $N_{K_\epsilon(r_i)} \subseteq C^t$ of K spatially nearest cells of r_i at t. The arbitrary selection of K does not affect the quality of the solution as long as it is high enough. We fix $capacity(l_j, r_i) = 0$ and

$$cost(l_j, r_i) = \alpha \, \|\phi(r_i) - \phi(l_j)\|_w \tag{5}$$

where $\phi(c) = [b(c)\, a(c)\, w(c)\, h(c)\, r(c)\, p(c)] \in \mathbb{R}^8$ is the feature vector associated to a cell c and α is a weighting parameter and w is the vector of weights given to the distances among the different features.

We continue formulating correspondence hypotheses for pairs of adjacent cells as the daughter cells of cellular mitosis events. Mitosis hypotheses are represented in the flow graph with the help of the vertices in S. For every pair of adjacent cells $r_i, r_j \in R$ a new vertex $s_{ij} \equiv s_{ji}$ is added to S to represent the union of r_i and r_j, adding arcs from s_{ij} to r_i and to r_j and setting $capacity(s, r_i) = 1$, $capacity(s, r_j) = 1$, $cost(s, r_j) = 0$ and $cost(s, r_j) = 1$. For each union of cells $s_{ij} \in S$ we formulate a set of K mitosis hypotheses each one respectively postulating that cells r_i and r_j are the daughter cells of the mitosis of cell l_k from the set $N_K\epsilon(s_{ij}) \subseteq C^t$ of K spatially nearest cells at time t of the union s_{ij} of cells r_i and r_j. We set $capacity(l_k, s_{ij}) = 0$ and

$$cost(l_k, s_{ij}) = \beta \, \|\phi(l_k) - \phi(r_i, r_j)\|_w \tag{6}$$

where $\phi(r_i, r_j)$ denotes the cell feature vector computed from the union of cells r_i and r, j and β is a weighting parameter. To ensure the flow constraint at s_{ij} it is necessary to add a link from A to s_{ij}, setting $capacity(A, s_{ij}) = 1$ and $cost(A, s_{ij}) = 0$.

Next we model hypotheses for cells leaving the scene at time $t + 1$. They are represented adding links from the cells to the auxiliary vertex D. Thus, for each cell $l_i \in L$ and arc is added from l_i to D. We set

$capacity(l_i, D) = 1$ and

$$cost(l_i, D) = \gamma \min_{x \in \text{Border}} \|B(l_i) - x\|_{w_b} \quad (7)$$

where Border denotes the set of points in the AJ graph perimeter and γ is a weighting parameter.

Hypotheses for apoptosis events are formulated in a similar way. For each vertex $l_i \in L$ we add an extra arc from l_i to D, we set capacity $capacity(l_i, D) = 1$ and

$$cost(l_i, D) = \delta\, a(l_i) \quad (8)$$

where δ is a weighting parameter. The cost of the hypothesis is proportional to the area of the cell as cells shrink during apoptosis. Note that we differentiate between cells leaving the scene or dying by apoptosis just by the edge selected in the solution to transport the flow from l_i to D.

Finally, we formulate hypotheses for cells entering the scene at time $t+1$. They are represented adding links from the auxiliary vertex A to the cells. Thus, for each cell $r_i \in R$ and arc is added from A to r_i. We set $capacity(A, r_i) = 1$ and

$$cost(A, r_i) = \epsilon\, min_{x \in \text{Border}} \|b(r_i) - x\|_{w_b} \quad (9)$$

where ϵ is a weighting parameter.

The parameters $\alpha \geq 0$, $\beta \geq 0$, $\gamma \geq 0$, $\delta \geq 0$ and $\epsilon \geq 0$ need to get proper values in order to obtain a successful solution to the correspondence problem. We restrict them such that $\alpha + \beta + \gamma + \delta + \epsilon = 1$. The solution to the correspondence problem is given by the amount of flow to be sent among each arc in the graph. The problem might be reformulated as an integer optimization problem as shown by [2]. The solution is then found employing branch and bound algorithm [3]. The correspondence among cells at frames t and $t+1$ is then recovered from the arcs selected in the solution.

Comparison of our cell segmentation method with the SeedWaterSegmenter method

We have performed a comparison of a 2D simplification of our cell segmentation method and the SeedWaterSegmenter method, an open source cell segmentation software available at [1]. With the aim of performing a fair comparison, we evaluate cell detection performance instead of AJ vertex and edge detection, as SeedWaterSegmenter has not been designed to solve this task.

The evaluation has been conducted in a similar way to the evaluation of vertex and edge detection, i.e., computing Precision, Recall and $F1$ measures from a wide variety of input parameters. SeedWaterSegmenter only depends on the width σ of the Gaussian kernel employing to smooth the images, while the 2D simplification of our method depends on 7 values: $V_{\sigma_{min}}$, $V_{\sigma_{max}}$, $P_{\sigma_{min}}$, $P_{\sigma_{max}}$, T_V, T_L and R_{max}. We consider a cell as a true detection if in the reference there is a cell centroid closer than $0.25\mu m$ to the detected one.

Figure S10 presents a Precision-Recall plot that we have obtained after testing the system with a broad range of input parameters processing the Notum dataset. The curve shows that the 2D simplification of our system outperforms the SeedWaterSegmenter in achieving higher precision and recall values. The experiment shows how the proposed approach is even able to perform well in a task it has not been specifically designed for. The main advantage of the SeedWaterSegmenter is that it requires adjustment of only one parameter, while our method requires adjustment of seven parameters.

[1] https://github.com/davidmashburn/SeedWaterSegmenter/

References

1. Mosaliganti K, Janoos F, Gelas A, Noche R, Obholzer N, et al. (2010) Anisotropic Plate Diffusion Filtering for Detection Cell Membranes in 3D Microscopy Images. In: Proceedings / IEEE International Symposium on Biomedical Imaging: from nano to macro. IEEE International Symposium on Biomedical Imaging. pp. 588–591. doi:10.1109/ISBI.2010.5490110.

2. Padfield D, Rittscher J, Roysam B (2011) Coupled minimum-cost flow cell tracking for high-throughput quantitative analysis. Medical Image Analysis 15: 650–668.

3. Nemhauser GL, Wolsey LA (1988) Integer and combinatorial optimization, volume 18. Wiley New York.
